# Highly efficient singlet oxygen generation, two-photon photodynamic therapy and melanoma ablation by rationally designed mitochondria-specific near-infrared AIEgens†

**DOI:** 10.1039/c9sc06441a

**Published:** 2020-01-21

**Authors:** Zheng Zheng, Haixiang Liu, Shaodong Zhai, Haoke Zhang, Guogang Shan, Ryan T. K. Kwok, Chao Ma, Herman H. Y. Sung, Ian D. Williams, Jacky W. Y. Lam, Kam Sing Wong, Xianglong Hu, Ben Zhong Tang

**Affiliations:** Department of Chemistry, Hong Kong Branch of Chinese National Engineering Research Center for Tissue Restoration and Reconstruction, Institute for Advanced Study, Department of Chemical and Biological Engineering, Institute of Molecular Functional Materials, Division of Life Science, State Key Laboratory of Molecular Neuroscience, The Hong Kong University of Science and Technology (HKUST) Clear Water Bay Kowloon Hong Kong China tangbenz@ust.hk; Department of Physics, HKUST Clear Water Bay Kowloon Hong Kong China; HKUST-Shenzhen Research Institute No. 9 Yuexing 1st RD, South Area, Hi-tech Park, Nanshan Shenzhen 518057 China; Center for Aggregation-Induced Emission, SCUT-HKUST Joint Research Institute, State Key Laboratory of Luminescent Materials and Devices, South China University of Technology Guangzhou 510640 China; MOE Key Laboratory of Laser Life Science, Institute of Laser Life Science, College of Biophotonics, South China Normal University 55 Zhongshan Avenue West Guangzhou 510631 China xlhu@scnu.edu.cn

## Abstract

Photosensitizers (PSs) with multiple characteristics, including efficient singlet oxygen (^1^O_2_) generation, cancer cell-selective accumulation and subsequent mitochondrial localization as well as near-infrared (NIR) excitation and bright NIR emission, are promising candidates for imaging-guided photodynamic therapy (PDT) but rarely concerned. Herein, a simple rational strategy, namely modulation of donor–acceptor (D–A) strength, for molecular engineering of mitochondria-targeting aggregation-induced emission (AIE) PSs with desirable characteristics including highly improved ^1^O_2_ generation efficiency, NIR emission (736 nm), high specificity to mitochondria, good biocompatibility, high brightness and superior photostability is demonstrated. Impressively, upon light irradiation, the optimal NIR AIE PS (DCQu) can generate ^1^O_2_ with efficiency much higher than those of commercially available PSs. The excellent two-photon absorption properties of DCQu allow two-photon fluorescence imaging of mitochondria and subsequent two-photon excited PDT. DCQu can selectively differentiate cancer cells from normal cells without the aid of extra targeting ligands. Upon ultralow-power light irradiation at 4.2 mW cm^−2^, *in situ* mitochondrial photodynamic activation to specifically damage cancer cells and efficient *in vivo* melanoma ablation are demonstrated, suggesting superior potency of the AIE PS in imaging-guided PDT with minimal side effects, which is promising for future precision medicine.

## Abstract 2

Mitochondria-targeted photosensitizers with highly efficient singlet oxygen generation, bright near-infrared AIE and good two-photon absorption are obtained through ingenious molecular engineering for cancer cell-selective photodynamic therapy.

## Graphical abstract

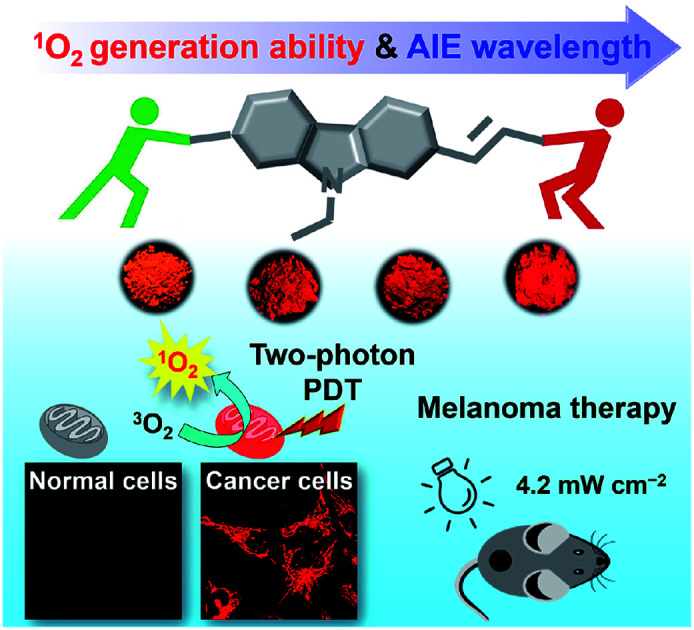

## Introduction

Cancer is a well-recognized major public health problem and will become a leading cause of morbidity and mortality in the coming decades worldwide.^1^ For example, there has been a steep rise in the number of patients with malignant melanoma. Malignant melanoma arises from melanocytes and is the most dangerous type of skin cancer with a poor prognosis in its latest stage due to the high propensity of tumor metastasis.^2–4^ Once melanoma spreads beyond its original location, its surgical treatment becomes extremely difficult and resistance to traditional chemotherapy and radiotherapy also dramatically increases.^5,6^ On the other hand, photodynamic therapy (PDT) is a promising alternative to these traditional treatments and is emerging as an effective modality with precise spatiotemporal control for cancer treatment due to its non-invasive character, few side effects, negligible drug resistance and low systemic toxicity.^7,8^ PDT relies on photosensitizers (PSs) and light to generate cytotoxic reactive oxygen species (ROS), particularly singlet oxygen (^1^O_2_), in the presence of oxygen to cause destruction of selected cells. With the above advantages, PDT has been approved in many countries for the treatment of lung, esophageal, bladder, skin, head and neck cancers.^9^ However, the clinical application of PDT is still far from ideal. The excess heating effect caused by strong laser irradiation, the intrinsic photo-toxicity and the low selectivity of PSs often result in harmful side-effects on healthy tissues.^8,10^ Excessive heating can be alleviated *via* the selection of a light source with low power density but for doing that PSs with high therapeutic efficacy are required. The selectivity of PDT can be realized by employing PSs that show preferred accumulation in tumor sites than healthy tissues. Moreover, it is useful to improve the targeting of PSs on the organelle level.^10^ As singlet oxygen has a very short lifetime (<40 ns) and a small action range (<20 nm), it is well accepted that mitochondria are the most ideal target organelles for therapeutic applications for their irreplaceable biofunctions in energy-generating and mediating cell death.^11–14^ Thus, it is promising for PSs to target mitochondria and *in situ* generate high dosage ^1^O_2_ to damage the mitochondria directly and activate programmed cell death. Such properties should potentially improve the therapeutic outcome in cancer therapy.^15–17^ As a result, PSs with efficient ^1^O_2_ generation, cancer cell selectivity and mitochondria-targeting capability are crucial to improve PDT efficacy.

Among diverse bioimaging techniques, fluorescence imaging has become a powerful tool for highly sensitive and noninvasive visualization of biological structures and processes in real time with high spatial resolution.^18^ Therefore, in terms of PSs, the coupling of efficient ^1^O_2_ generation with bright emission has been used for imaging-guided PDT, which has emerged as a promising alternative for cancer treatment.^19,20^ An ideal PS for imaging-guided PDT should possess characteristics including strong near-infrared (NIR) (>700 nm) absorption, bright NIR emission, highly efficient ^1^O_2_ generation, negligible dark toxicity, good photostability and biocompatibility.^21^ Actually, a number of organic NIR fluorophores such as porphyrin, chlorin, phthalocyanine and BODIPY derivatives have been recognized as PSs for imaging-guided PDT.^22,23^ However, these PSs often suffer from several intrinsic drawbacks including small Stokes' shift, low fluorescence quantum yield, moderate ^1^O_2_ production, nonspecific targeting capability, poor photostability and unsatisfied biocompatibility.^24^ Even more, these conventional PSs often possess a rigid planar π-conjugated structure and are prone to aggregate in aqueous media, resulting in emission quenching notoriously known as aggregation-caused quenching (ACQ) and insufficient ^1^O_2_ production, both of which constitute other critical obstacles of conventional PSs for imaging-guided PDT.^24,25^

The discovery of a new emerging type of PSs with aggregation-induced emission (AIE) characteristics has solved the issues discussed above.^26^ AIE is exactly opposite of ACQ and was originally proposed by Tang in 2001.^27–29^ The restriction of intramolecular motion (RIM) was identified as the main working mechanism.^30^ AIE luminogens (AIEgens) have been demonstrated as promising alternatives to traditional ACQ fluorophores for fluorescence imaging because of their stronger emission in aggregates, larger Stokes' shift, superior photostability and high potential to develop “wash-free” and “light-up” probes.^31–36^ More impressively, recent studies pointed out that AIEgens could also efficiently generate ^1^O_2_ in the aggregate state, making them promising as PSs for image-guided PDT.^26,37^ However, most of the current AIE PSs display absorption and emission at short wavelengths. Thus, the development of AIE PSs with both excitation and emission in the NIR region is in great demand for biological applications because of the advantages of lesser photodamage to cells, lower scattering, deep light penetration and lower interference from tissue autofluorescence.^38,39^ Two-photon excitation is an effective way to translate the excitation wavelength of AIEgens into the NIR region and shows additional advantage in terms of high spatial resolution due to its intrinsic confocal nature.^40^ Fabrication of NIR AIEgens with excellent two-photon absorption properties is especially important for optical *in vivo* deep imaging and therapy.^41^ Moreover, the efficiency of ^1^O_2_ generation needs to be improved for a high therapeutic effect. Therefore, AIE PSs with NIR two-photon excitation, bright NIR emission, efficient ^1^O_2_ generation, cancer cell selectivity and mitochondrial specificity are significantly favorable for imaging-guided PDT but rarely concerned.

In this work, a series of AIEgens based on positively charged pyridinium and quinolinium moieties with different donor–acceptor (D–A) strength were developed. The modulation of D–A strength within AIEgens is demonstrated to be an effective strategy to simultaneously improve ^1^O_2_ generation and red shift the emission. Among the synthesized molecules, DCQu displays extremely high ^1^O_2_ generation efficiency, bright NIR emission and NIR two-photon activity. DCQu can not only selectively target cancer cells over normal cells but also specifically stain mitochondria with good photostability. Both *in vitro* cancer cell-selective ablation and *in vivo* melanoma therapy under one- or two-photon excitation suggest the superior performances of DCQu in imaging-guided PDT.

## Results and discussion

**Fig. 1:**
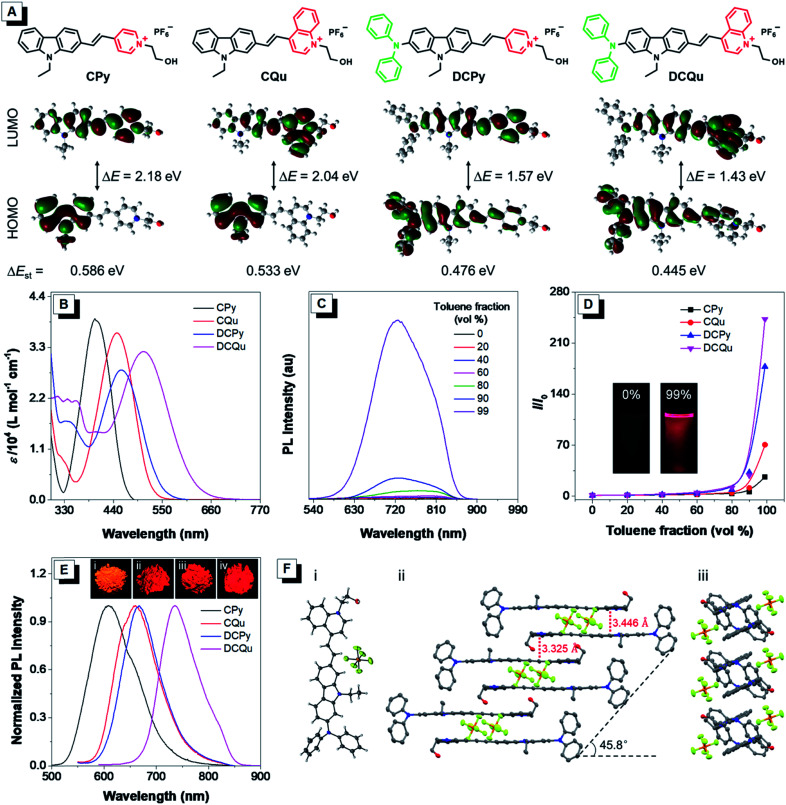
(A) Molecular structures of AIEgens (CPy, CQu, DCPy and DCQu) and their molecular orbital amplitude plots of HOMOs and LUMOs calculated by TD-DFT at the B3LYP/6-31G (d,p) basis set. (B) Absorption spectra of CPy, CQu, DCPy and DCQu in DMSO. (C) PL spectra of DCQu in DMSO/toluene mixtures with different toluene fractions. [DCQu] = 15 μM, *λ*_ex_ = 500 nm. (D) Plot of relative PL intensity (*I*/*I*_0_) *vs.* the composition of the DMSO/toluene mixtures of CPy, CQu, DCPy and DCQu, where *I*_0_ = PL intensity in pure DMSO solution. Inset: fluorescent photographs of DCQu in the DMSO solution and in a DMSO/toluene mixture with 99% toluene fraction taken under 365 nm UV irradiation. (E) Normalized PL spectra of the fluorogens in the solid state. Inset: fluorescence photographs of (i) CPy, (ii) CQu and (iii) DCPy taken under 365 nm UV irradiation and (iv) DCQu taken using a CRI *in vivo* imaging system. (F) (i) Single-crystal structure of DCQu. (ii) Molecular stacking structure along the long molecular axis and (iii) the short molecular axis. The hydrogen atoms in (ii) and (iii) are omitted for clarity.

One of the key protocols to improve the photosensitization of PSs to favor ^1^O_2_ generation is to enhance the intersystem crossing (ISC) from the lowest excited singlet state (S_1_) to the lowest triplet state (T_1_).^26^ Previous studies demonstrated that the ISC rate can be effectively elevated by the introduction of heavy atoms. Unfortunately, this will generally lead to dark toxicity to PSs.^42^ Recently, an alternatively strategy was proposed to improve the ISC by decreasing the energy gap (Δ*E*_st_) between S_1_ and T_1_ based on the separation of the orbital distribution of the highest occupied molecular orbitals (HOMOs) and the lowest unoccupied molecular orbitals (LUMOs).^43^ Structurally, the enhancement of donor–acceptor (D–A) strength of a fluorophore favors the above mentioned separation and ISC process, facilitating ^1^O_2_ generation for efficient PDT.^37^ On the other hand, strong D–A strength of a fluorophore could also facilitate intramolecular charge transfer (ICT) to lead to small electronic bandgaps and long absorption and emission wavelengths. Besides the electron-withdrawing potency, the introduction of electron acceptors with positive charge could probably endow fluorophores with unique biological functions of selective cancer cell accumulation and subsequent mitochondrial localization within cancer cells.^28,44^ The D–A effect together with extended π-conjugation could also improve the two-photon absorption (2PA) properties of fluorophores, and translate the excitation wavelength to the NIR region.^45^ To verify these hypotheses, our design started from CPy comprising a pyridinium moiety as an acceptor and a carbazole fragment as a donor and a π-bridge (Fig. 1A). Further molecular engineering of the fluorophore structure was performed by replacing the pyridinium moiety with a stronger electron-accepting quinolinium salt or/and introducing an electron-donating diphenylamine at the end of carbazole, yielding fluorogens named CQu, DCPy and DCQu with different D–A strength. Four fluorogens were synthesized by typical Knoevenagel condensation in high yields and fully characterized (Scheme S1, Fig. S1–S23, Tables S1 and S2†). Prior to the optical studies, density functional theory (DFT) calculations of the new molecules were performed to understand their ICT. As shown in Fig. 1A, the electron clouds of HOMOs of CPy and CQu, and DCPy and DCQu were mainly located on the carbazole ring, and diphenylamino and carbazole frameworks, respectively. The LUMOs of all compounds, however, were primarily contributed by the orbitals of the acceptor moiety and partially the carbazole ring. The calculated band gaps (Δ*E*) of CPy, CQu, DCPy and DCQu were 2.18, 2.04, 1.57 and 1.43 eV, respectively, suggesting a gradual enhancement of the ICT effect. Accordingly, the Δ*E*_st_ values were calculated to be 0.586, 0.533, 0.476 and 0.445 eV for CPy, CQu, DCPy and DCQu, respectively, showing a gradual decrease in Δ*E*_st_.

The photophysical properties of the new fluorogens were subsequently investigated by UV-vis absorption and photoluminescence (PL) spectroscopy. As depicted in Fig. 1B, CPy showed an absorption maximum at 398 nm in dimethylsulfoxide (DMSO) attributed to ICT from the carbazole moiety to the pyridinium group. The replacement of the pyridinium group with a stronger electron-withdrawing quinolinium unit or the introduction of a strong electron-donating diphenylamine moiety on the carbazole ring both could red shift the absorption of CQu and DCPy to 448 and 458, respectively, again due to the increased ICT effect. In contrast, DCQu with diphenylamine and quinolinium moieties attached at two ends of the carbazole ring displayed further red-shifted and broadened absorption with a maximum at 507 nm, indicating a stronger ICT effect. A gradual bathochromic shift in the absorption of fluorophores from CPy to CQu, then DCPy and DCQu was nicely consistent with the theoretical results discussed above.

The AIE properties of CPy, CQu, DCPy and DCQu were studied in DMSO/toluene mixtures with different toluene fractions (Fig. 1C, D and S24†). All the compounds showed very weak emission in DMSO with 1.4%, 0.4%, 0.9% and 0.3% of quantum yields for CPy, CQu, DCPy and DCQu, respectively, due to the active intramolecular motions, which consumed the energy of the excited state through nonradiative pathways. Upon gradual addition of toluene to the DMSO solution, the fluorescence of the molecules became stronger. At 99 vol% toluene fraction, the fluorescence intensity of CPy, CQu, DCPy and DCQu was 27, 70, 177 and 243-fold, respectively, higher than that in the pure DMSO solution, due to the RIM mechanism. These results demonstrated that CPy, CQu, DCPy and DCQu were AIE active and emitted yellow to NIR light at 597, 615, 659 and 725 nm, respectively. Owing to the AIE characteristics, CPy, CQu, DCPy and DCQu showed bright solid-state fluorescence at 609, 660, 667 and 736 nm with fluorescence quantum yields of 15.1%, 5.2%, 3.8% and 3.2%, respectively, determined by an integrating sphere (Fig. 1E). The above results also revealed that the emission color of these fluorophores could be facilely extended to the NIR region by modulating the ICT strength. Time-resolved fluorescence measurements of CPy, CQu, DCPy and DCQu in the solid state revealed lifetimes of 5.64, 6.28, 2.64 and 1.34 ns, respectively (Fig. S25†). Moreover, all the compounds showed very large Stokes Shifts (Δ*ν* = 209–229 nm), which could be attributed to an excited-state intramolecular charge transfer between the electron donor and the electron acceptor within the same dye molecule.^46,47^ The large Stokes shifts were favorable for bio-imaging due to the little interference between excitation and emission. The absorption and emission properties of DCQu in different solvents with varied polarities were investigated. As shown in Fig. S26,† DCQu showed very weak fluorescence in various organic solvents.

The AIE effect and bright NIR solid-state fluorescence of the compounds trigger us to elucidate their molecular conformation and molecular arrangement in the solid state. Single crystals of DCQu were obtained by slow evaporation from its ethanol/CH_2_Cl_2_ mixture and characterized by X-ray crystallography. The crystal data and collection parameters are summarized in Table S2.† DCQu crystallized in the monoclinic *P*2_1_/*c* space group with four molecules in an elemental cell. DCQu was found to adopt a *trans*-conformation (Fig. 1F). In the crystal structure, the small dihedral angle of 1.65° between the carbazole and the quinolinium moieties suggested that they were essentially planar and conjugated to allow good π-electron delocalization of the whole molecule. Thus, strong push–pull character in combination with the extended π-conjugation within the fluorophore effectively shifted its emission to the NIR region. As shown in Fig. 1F, the planar molecules further arranged into offset columnar stacks of antiparallel dimers along the long molecular axis with a slip angle of 45.8°. This revealed a typical J-type packing through close intermolecular π–π stacking. Besides, the crystal packing diagrams of DCQu showed that multiple inter- and intramolecular interactions such as P–F⋯H, C–H⋯π and π⋯π interactions were present in the crystal lattice, which could rigidify the molecular conformation and lock the intramolecular rotations. Thus, the energy consumption of the excited state by intramolecular rotation was greatly reduced in the solid state, enabling the AIEgen molecules to emit intense NIR emission.

The integration of AIE characteristics and large Stokes shift makes CPy, CQu, DCPy and DCQu promising candidates for biological applications. Cell imaging experiments were initially conducted by incubating HeLa cells with different concentrations of DCQu for 15, 30 and 60 min respectively, followed by CLSM imaging excited at 488 nm (Fig. S27†). Both concentration and incubation time had an obvious influence on the cell imaging. For the same incubation time, the fluorescence signal gradually increased with the content improvement of DCQu. On the other hand, for cells incubated with DCQu with the same content, the fluorescence signal also enhanced with the extension of the incubation time from 15 to 30 min, while further increasing the culture time to 60 min, it didn't cause an obvious difference in the fluorescence signal. Thus, the following cell imaging experiments were performed with a relatively low content of probes at 1 μM, incubating for 30 min. Notably, the fluorescence of DCQu could still be observed in cells upon incubation with DCQu as low as 0.1 μM, further suggesting high brightness of DCQu in cell imaging.

**Fig. 2:**
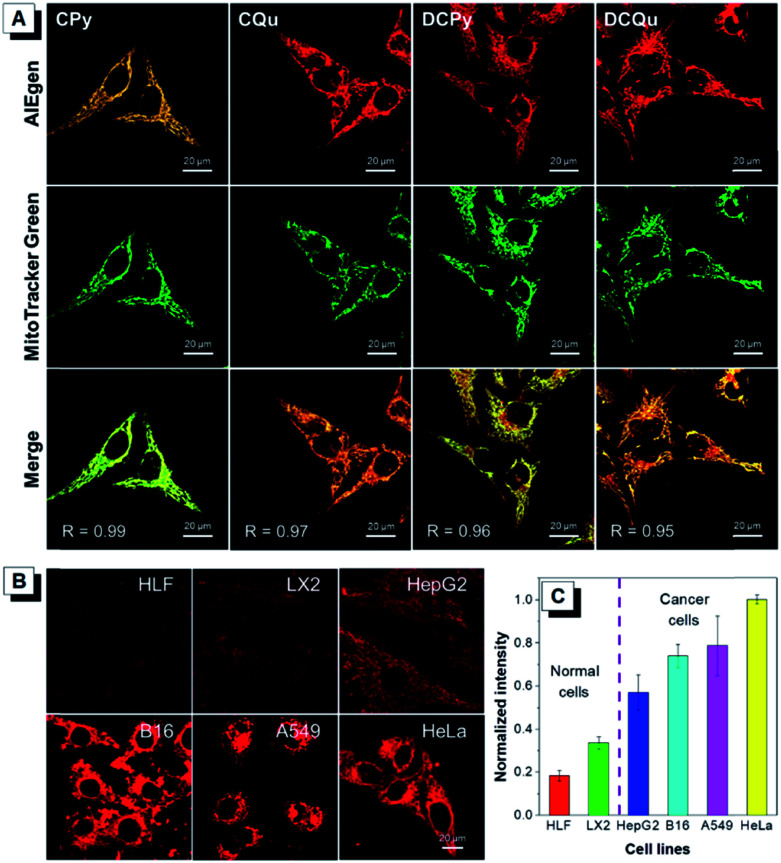
(A) Co-localization images of HeLa cells stained with CPy, CQu, DCPy and DCQu and MitoTracker Green, and Merged images. The displayed Pearson's correlation coefficient (*R*) denotes the goodness of colocalization. [AIEgens] = 1 μM, [MitoTracker Green] = 0.5 μM, and *λ*_ex_ = 488 nm. Scale bar = 20 μm. (B) Fluorescence images of different normal cells (HLF and LX2) and cancer cells (HepG2, B16, A549 and HeLa) stained with DCQu for 30 min. [DCQu] = 1 μM. (C) Relative fluorescence intensity of different cells incubated with DCQu for 30 min. The intensity data were measured by image J. [DCQu] = 1 μM.

To further investigate the specificity of these AIEgens for cell imaging, typical colocalization experiments were carried out by incubating HeLa cells with each AIEgen and then co-stained with MitoTracker Green, which was a commercially available probe for mitochondrial localization (Fig. 2A). The red channel of each AIEgen perfectly overlapped with that of MitoTracker Green, which exhibited high Pearson's correlation coefficients of 0.99, 0.97, 0.96 and 0.95 for CPy, CQu, DCPy and DCQu, respectively, indicating the superior specificity of the designed AIEgens for mitochondrial staining. The mitochondrial targeting capability of the cationic lipophilic AIEgens mainly relied on the driving force of a very large membrane potential of around 180 mV across the mitochondrial membrane.^48–50^ AIEgen may be uptaken by cells through endocytosis in the aggregate form or may diffuse into cells in the isolated form. Even isolated AIEgen can specifically light up the mitochondria and the fluorescence signal will become stronger and stronger upon aggregate formation by concentration accumulation. Photostability, as a key criterion for evaluating a fluorescent bioprobe, of four AIEgens was subsequently checked by continuous laser excitation and sequential scanning with a confocal microscope (Fig. S28†). These results demonstrated that all four AIEgens showed excellent photostability, which was even better than that of MitoTracker Green.

Recent studies have demonstrated that cancer cells generally possess a more negatively charged surface than normal cells, because the positive ions on the cancer cell surface can be removed by the secreted lactate anions generated by the higher level of lactate secretion in the elevated glycolysis of cancer cells.^51^ Besides, the more active metabolism of cancer cells shows a higher mitochondrial membrane potential (MMP) than normal cells with a difference of at least 60 mV. This unique electrostatic pattern on the cancer cell membrane and their mitochondrial membrane has been proven to be a powerful driving force to discriminate cancer cells over normal cells through the strong electrostatic interaction with a positively charged object.^44,49^ The intrinsic positive charge and mitochondria-specific capability as well as the high ^1^O_2_ generation efficiency and the NIR AIE properties of DCQu therefore inspired us to investigate its differentiation of cancer cells from normal cells. Thus, various cancer cells and normal cells were incubated with DCQu under the same conditions, followed by CLSM observation (Fig. 2B and S29†). DCQu was more prone to accumulate within cancer cells, including HepG2, B16, A549 and HeLa cells, and subsequently stained the mitochondria with high brightness and a high signal-to-noise ratio. The difference in emission intensity may be attributed to the different mitochondrial membrane potential for different cancer cells.^52–54^ By contrast, normal cells such as HLF and LX2 displayed much weaker fluorescence, which was also quantificationally evaluated (Fig. 2C). These results demonstrated a promising capability of DCQu for selective cancer cell targeting without using any biomarkers or extra modification of targeting ligands.

Notably, mitochondria, one of the most important intracellular organelles responsible for cellular energy production, are crucial in regulating cell apoptosis. The direct damage and destruction of mitochondria would initiate programmed cell death efficiently.^55^ However, for traditional PDT application, the long diffusion distance of ^1^O_2_ to mitochondria probably weakens the damage extent towards cancer cells due to the high reactivity and a short lifetime of ^1^O_2_.^56^ Thus, it would be ideal for improving therapeutic efficiency if PSs can be localized at mitochondria and subsequently cause damage *in situ* by the generated ^1^O_2_ with light irradiation, which was recently realized by designing a complicated macromolecular protein–ruthenium complex.^57^ With this in mind, the ^1^O_2_ generation efficiency of four AIEgens was subsequently investigated.

**Fig. 3:**
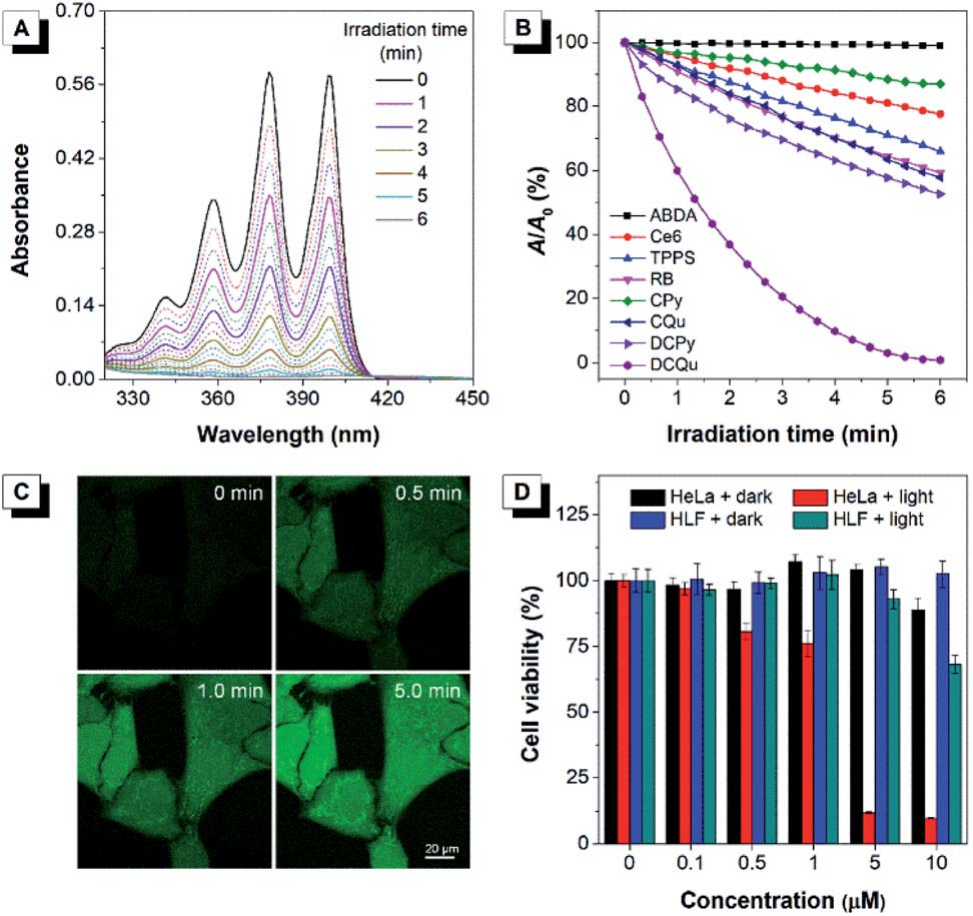
(A) Absorption spectra of ABDA in the presence of DCQu under white light (4.2 mW cm^−2^) irradiation. [DCQu] = 5 μM, [ABDA] = 50 μM, and the time interval for UV measurement = 20 s. (B) Decomposition of ABDA in the presence of different PSs under light irradiation, where *A*_0_ and *A* are the absorbance of ABDA at 378 nm before and after irradiation. [PSs] = 5 μM, [ABDA] = 50 μM, and the time interval for UV measurement = 20 s. (C) Detection of intracellular ROS generation using H2DCF-DA in HeLa cells incubated with DCQu followed by irradiation with white light irradiation for different times. (D) Cell viability of HeLa cancer cells and HLF normal cells stained with different concentrations of DCQu in the absence or presence of white light irradiation.

Considering that all these AIEgens had strong absorption in the visible light region, the ^1^O_2_ generation ability of AIEgens was initially evaluated under ultralow-power white light irradiation (400–700 nm, 4.2 mW cm^−2^) using a commercial ^1^O_2_ indicator, 9,10-anthracenediyl-bis(methylene)-dimalonic acid (ABDA), which could undergo oxidation by ^1^O_2_ to display a gradual decrease in absorption (Fig. 3A). Under white light irradiation, the absorbance of the ABDA solution in the presence of CPy decreased slowly with the extension of the irradiation time (Fig. 3B). For CQu and DCPy with a stronger ICT effect, the degradation of ABDA was much faster than that of CPy and even slightly faster than that of Ce6. By contrast, the absorbance of the ABDA solution in the presence of DCQu decreased dramatically with time; specifically 100 nmol of ABDA was completely consumed by 10 nmol of DCQu at 6 min (Fig. 3A and B), at which only 13.1, 42.2 and 47.4 nmol of ABDA were degraded by CPy, CQu and DCPy, respectively. Thus, the ^1^O_2_ generation efficiency of DCQu was 7.6, 2.4 and 2.1-fold higher than those of CPy, CQu and DCPy, respectively. These results demonstrated that the enhancement in ^1^O_2_ generation efficiency was gradually realized by the step-by-step molecular engineering in the order of CPy, CQu, DCPy and DCQu, which was nicely consistent with their gradually decreased Δ*E*_st_ in the order of 0.586, 0.533, 0.476 and 0.445 eV for CPy, CQu, DCPy and DCQu, respectively. For DCQu, the highest efficiency of ^1^O_2_ generation in the series was benefited from its smallest value of Δ*E*_st_ resulting from strong D–A strength. The resulting small Δ*E*_st_ could facilitate the ISC process from S_1_ to T_1_ and result in a considerable improvement in the yield of the triplet excited state, facilitating the ^1^O_2_ generation. Furthermore, three well-known and mostly used PSs, including Ce6, TPPS and Rose Bengal (RB), with high ^1^O_2_ generation efficiencies only consumed 22.5, 34.1, and 40.7 nmol of ABDA under the same conditions, respectively, indicating that the ^1^O_2_ generation ability of DCQu was 4.4, 2.9 and 2.5-fold stronger than those of Ce6, TPPS and Rose Bengal, respectively (Fig. 3B). To the best of our knowledge, the ^1^O_2_ generation ability of DCQu was much superior to that of previously reported AIE PSs.^26^ We then evaluated the white light irradiation-activated ROS generation of DCQu inside HeLa cells through incubation either with both H2DCF-DA and DCQu or with H2DCF-DA alone. An obvious increase in the fluorescence signal was observed from the cells incubated with both H2DCF-DA and DCQu with increasing irradiation time, revealing efficient ROS generation for DCQu over the course of irradiation (Fig. 3C). In contrast, no obvious fluorescence increase was observed in the absence of DCQu (Fig. S30†). Then, the mitochondrial changes of HeLa cells stained with DCQu were monitored by continuous scanning under CLSM (Fig. S31†). Upon increasing the scanning time, the reticulum-like mitochondrial structures were gradually fragmented and granulated, which will lead to cell death eventually. Furthermore, the Annexin V-FITC assay was performed after the PDT treatment for detecting cell apoptosis.^58,59^ Obvious cell apoptosis caused by DCQu under light with green PL in the cell membrane was observed, while the cells without light irradiation showed no obvious apoptotic behaviour (Fig. S32†).

Apparently, the rationally designed DCQu was an ideal PS candidate possessing strong ^1^O_2_ generation, cancer cell selective accumulation and subsequent mitochondria-specific targeting capabilities, all of which motivated us to further investigate its performance in cancer cell selective and mitochondrial targeting PDT. Then, *in vitro* quantitative evaluation was performed by the standard MTT assay against HeLa cancer cells (Fig. 3D). Upon incubation with DCQu under dark conditions, the cell viability was still higher than 89%, despite increasing the concentration of DCQu up to 10 μM, suggesting minimal cytotoxicity of DCQu under dark conditions. However, along with the white light irradiation, DCQu displayed a remarkable dose-dependent toxicity, the cell viability decreased as low as 9% at 10 μM, thus DCQu demonstrated a promising capability for cancer cell ablation through the photodynamic process. On the other hand, to further verify the selectivity of DCQu in killing cancer cells over normal cells, the evaluation of dose-dependent cytotoxicity was conducted under the same conditions employing HLF cells as a normal cell line. As a result, DCQu displayed negligible dark cytotoxicity on HLF cells, which was similar to that on HeLa cells, however, under same light irradiation, the cell viability slightly decreased to 68% at a concentration of 10 μM, revealing much less extent damage to normal cells than cancer cells, which mainly benefited from much more significant accumulation of DCQu in cancer cells than normal cells. These results suggested that DCQu with cancer cell-specific staining and subsequent photodynamic killing capability has great potential for precision cancer theranostics due to its intrinsic targeting potency.

**Fig. 4:**
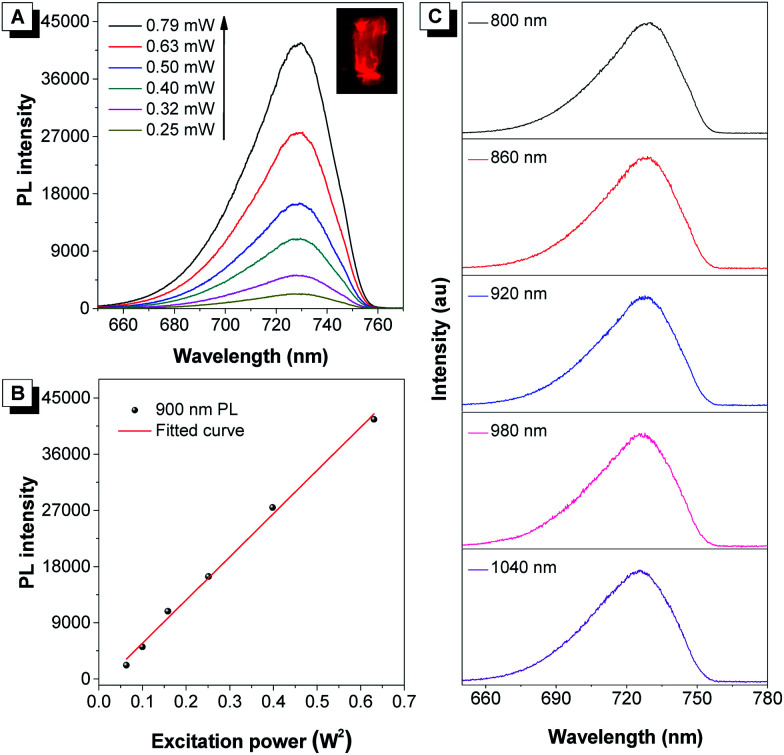
(A) The upconversion PL spectra of crystalline powder of DCQu at different input powers at 900 nm. Inset: Fluorescent photograph of DCQu crystals taken under a fluorescence microscope. (B) The corresponding linear relationship between the output fluorescence intensity and the square of input laser power (*W*^2^). (C) The two-photon excitation window of crystalline powder of DCQu.

It was demonstrated that DCQu displayed high ^1^O_2_ generation efficiency, AIE-active NIR emission, excellent photostability and efficient one-photon *in vitro* PDT efficiency. The strong push–pull dipolar character and the extended conjugation of DCQu were expected to endow the molecule with 2PA properties, allowing image-guided two-photon photodynamic therapy. In view of its strong solid-state fluorescence, the two-photon-excited fluorescence of DCQu was studied in the solid state. The upconversion PL spectrum of DCQu obtained at 900 nm laser light excitation in the solid state resembled that measured under one-photon conditions with a similar emission maximum, illustrating that the emission processes from the one- and two-photon excited states to the ground state were consistent (Fig. 4A). When the power of the excitation source was increased, the two-photon-excited fluorescence intensity showed a square dependence on the incident energy, implying that upconverted emission stemmed from a 2PA process (Fig. 4B). Additionally, DCQu presented a broad two-photon excitation window ranging from 800 nm to 1040 nm in the solid state (Fig. 4C). The 2PA cross-section of DCQu in dioxane at 900 nm was calculated to be 95 GM using rhodamine B as the reference. These results demonstrated the design of the conjugated dipolar AIEgen to definitely achieve good 2PA located at the biological transparency window.

**Fig. 5:**
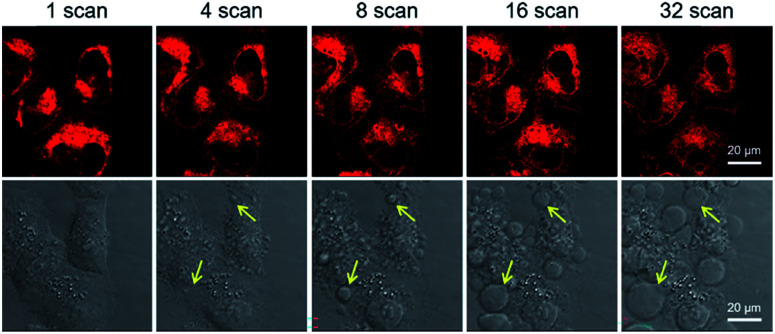
Two-photon excited fluorescence (top row) and bright-field (bottom row) images of HeLa cells stained with DCQu (5 μM) followed by different two-photon (900 nm, fs Ti:sapphire laser) scans.

Next, the applicability of DCQu for two-photon imaging of mitochondria was investigated. DCQu could clearly visualize the mitochondria within HeLa cells under two-photon excitation at 900 nm, revealing that DCQu is a promising candidate as a two-photon imaging probe to achieve NIR-to-NIR mitochondrial imaging in living cells (Fig. S33†). Furthermore, to evaluate the therapeutic effect of DCQu under two-photon excitation, HeLa cells stained with DCQu were irradiated with different 900 nm two-photon fs laser scans. The increase of two-photon scans caused a gradual and significant change of cell morphology, generating obvious bubbles upon increasing the irradiation scan number (yellow arrows indicated) (Fig. 5). These changes were associated with cell death, which was apparently initiated by the PDT treatment of DCQu under the two-photon excitation, revealing its great potency for mitochondria-targeted two-photon PDT.^60^

**Fig. 6:**
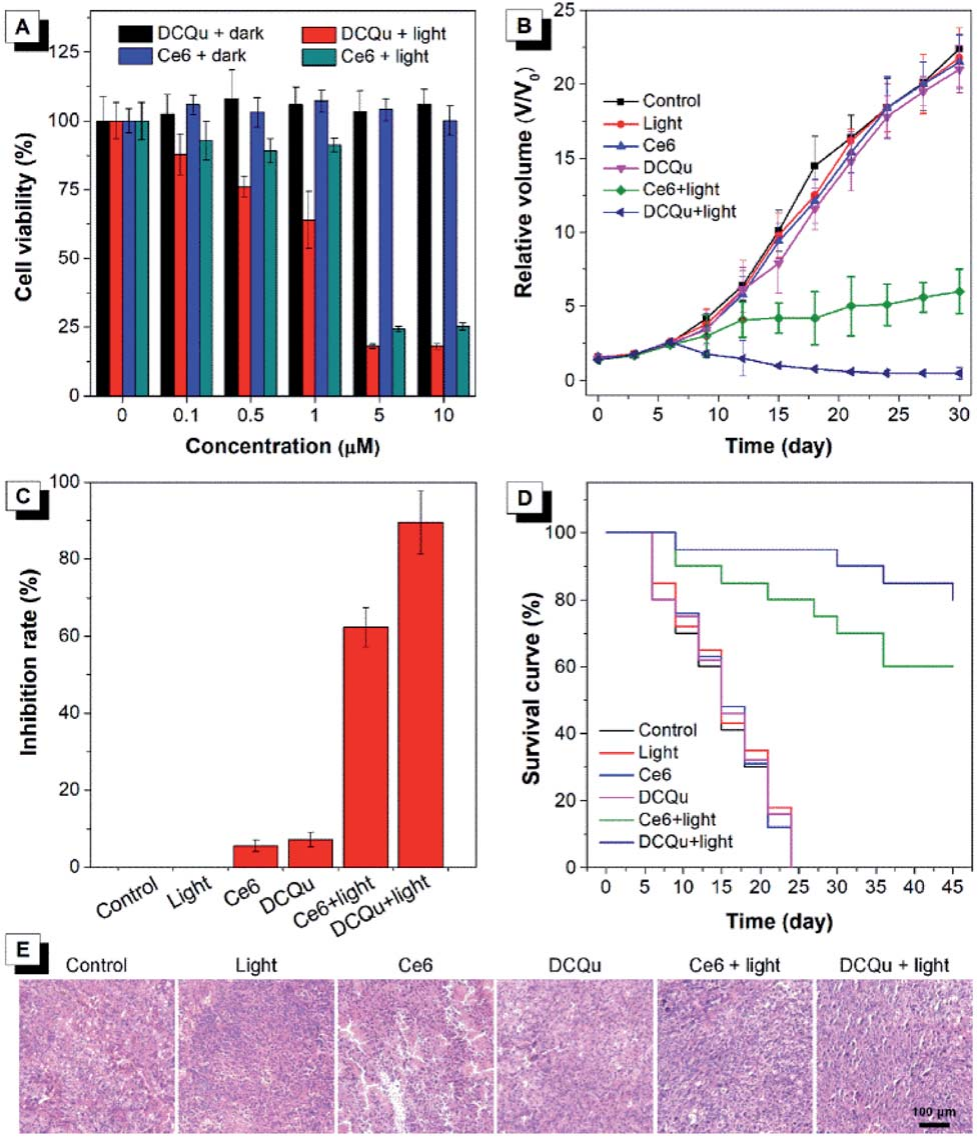
(A) Cell viability of melanoma cancer B16 stained with different concentrations of DCQu or Ce6 in the absence or presence of white light irradiation. (B) Tumor growth curves of B16 melanoma-bearing mice after different treatments. (C) Calculated tumor inhibition ratios of each treatment group. (D) Survival rate of mice after different treatments. (E) Histological sections of tumor tissues stained with hematoxylin and eosin. Scale bar = 100 μm.

The superior performance in *in vitro* PDT makes DCQu a promising PS for *in vivo* PDT applications. Melanoma is one of the most dangerous skin cancers and quite suitable for PDT treatment.^61–63^ Herein, a typical mouse melanoma model was constructed for evaluating the *in vivo* PDT applications of DCQu. Prior to *in vivo* experiment, *in vitro* cell inhibition against B16 cells was evaluated and further compared with that of Ce6 (Fig. 6A). Upon white light irradiation, significant cell inhibition was observed for DCQu reflected by the gradual decrease in cell viability to 18% at 5 μM, at which 24% of cell viability was obtained for Ce6, revealing a much better cancer cell inhibition induced by DCQu than that of Ce6. For *in vivo* PDT evaluation of DCQu on B16-bearing mice, mice were randomly divided into 6 groups, including (1) PBS (control), (2) light irradiation, (3) Ce6 at dark, (4) DCQu at dark, (5) Ce6 with light irradiation, and (6) DCQu with light irradiation, respectively. The light irradiation was performed using a LED light with an ultralow power density of 4.2 mW cm^−2^. Typical intratumoral treatments were performed every three days in one month (Fig. S34 and S35†). The regression efficacy in tumor growth was investigated to evaluate the therapeutic efficiency of DCQu (Fig. 6B). Almost negligible inhibition of tumor growth was observed for the mice treated with light, Ce6 or DCQu alone, as compared with the control group, indicating that pure light irradiation or PS in the dark did not cause any antitumor effect. Obviously, compared to rapid tumor growth in the control group within 30 d, commercial Ce6 could gradually inhibit tumor growth under light irradiation in 30 day treatments, but the tumor volume increased moderately in terms of the relative tumor volume. Dramatically, just three successive treatments in the group of “DCQu + light” effectively stopped the tumor growth trend, and further treatments significantly shrinked the tumor size to a minimum value starting from day 24, and the tumor was even eradicated and vanished in most of the treated mice. Remarkably, DCQu together with light irradiation gave rise to a high tumor inhibition rate of 89.5%, which was much higher than that of 62.3% for Ce6 (Fig. 6C). Impressively, almost complete cure of melanoma was observed for the mice treated with DCQu under light irradiation (Fig. 6B and C). These results demonstrated much higher PDT therapeutic efficiency of DCQu than that of Ce6 in tumor inhibition, even with an ultralow irradiation power of 4.2 mW cm^−2^.

Furthermore, the survival percentages of mice after diverse treatments were analysed accordingly (Fig. 6D). The survival percentages of the control group and the mice treated with only light irradiation, Ce6 or DCQu without light irradiation all fell rapidly in one month. In contrast, the survival percentage of “Ce6 + light” or “DCQu + light”-treated mice remained to be ∼60% and ∼80%, respectively at day 45, indicating that the PDT application of DCQu significantly inhibited tumor growth and prolonged the survival life of these tumor-bearing mice. Furthermore, H&E analysis revealed that the group of “DCQu + light” exhibited much more pyknotic cells with highly condensed nuclei, which were apoptotic or dead cells (Fig. 6E). Noteworthily, there was no obvious body weight change during the whole PDT treatments (Fig. S36†), and undetectable pathological abnormality was observed for main organs by H&E staining (Fig. S37†), further corroborating the negligible side effects and the excellent biocompatibility of DCQu itself and the PDT treatment processes.

## Conclusions

In this work, a simple rational strategy for the molecular engineering of mitochondria-targeting PSs is performed, affording an optimized DCQu with desirable concerted advantages of highly efficient ^1^O_2_ generation, intense NIR emission (736 nm), NIR two-photon excitation, AIE characteristics and a large Stokes' shift (229 nm). DCQu could differentiate cancer cells over normal cells and further specifically illuminate mitochondria of cancer cells under both one- and two-photon excitation, which is favorable for highly specific photodynamic killing of cancer cells rather than normal cells by both one- and two-photon PDT. Impressively, the ^1^O_2_ generation capability of DCQu is extraordinarily higher than that of popularly commercial PSs, including Ce6, TPPS and Rose Bengal. Both *in vitro* cancer cell selective ablation and *in vivo* melanoma therapy demonstrate the superior inhibition efficiency of DCQu under an ultralow-power lamp light of 4.2 mW cm^−2^ with excellent biocompatibility and minimal potential side effects. Synergistic optimization of the PDT treatment based on the NIR 2PA, NIR AIE and efficient ^1^O_2_ generation of DCQu was expected to treat pathogen infection and other diseases in future biomedicine. It is believed that the successful example of molecular design of AIE-active PSs presented in this work will stimulate the development of more efficient PSs for potential translational medicine.

## Ethical statement

All animal procedures were performed following the Guidelines for Care and Use of Laboratory Animals of South China Normal University and approved by the Animal Ethics Committee of South China Normal University.

## Conflicts of interest

There are no conflicts to declare.

## Supplementary Material

SC-011-C9SC06441A-s001

SC-011-C9SC06441A-s002
