# Supplementary material for: Highly efficient singlet oxygen generation, two-photon photodynamic therapy and melanoma ablation by rationally designed mitochondria-specific near-infrared AIEgens
Source: Chem Sci. 2020 Jan 21;11(9):2494–503. doi: 10.1039/c9sc06441a (PMC8157451; doi:10.1039/c9sc06441a)
Supplement: SC-011-C9SC06441A-s001 [file SC-011-C9SC06441A-s001.pdf]

Electronic Supplementary Material (ESI) for Chemical Science.
This journal is © The Royal Society of Chemistry 2019

Electronic Supplementary Information

Highly efficient singlet oxygen generation, two-photon photodynamic therapy and melanoma ablation by rationally designed mitochondria-specific near-infrared AIEgens†

Zheng Zheng,‡[a] Haixiang Liu,‡[a] Shaodong Zhai,[e] Haoke Zhang,[a] Guogang Shan,[a] Ryan T. K. Kwok,[a] Chao Ma,[b] Herman H. Y. Sung,[a] Ian D. Williams,[a] Jacky W. Y. Lam,[a] Kam Sing Wong,[b] Xianglong Hu*[e] and Ben Zhong Tang*[acd]

[a.] Department of Chemistry, Hong Kong Branch of Chinese National Engineering Research Center for Tissue Restoration and Reconstruction, Institute for Advanced Study, Department of Chemical and Biological Engineering, Institute of Molecular Functional Materials, Division of Life Science and State Key Laboratory of Molecular Neuroscience, The Hong Kong University of Science and Technology (HKUST), Clear Water Bay, Kowloon, Hong Kong, China. E-mail: tangbenz@ust.hk
[b.] Department of Physics, HKUST, Clear Water Bay, Kowloon, Hong Kong, China
[c.] HKUST-Shenzhen Research Institute, No. 9 Yuexing 1st RD, South Area, Hi-tech Park, Nanshan, Shenzhen 518057, China
[d.] Center for Aggregation-Induced Emission, SCUT-HKUST Joint Research Institute, State Key Laboratory of Luminescent Materials and Devices, South China University of Technology, Guangzhou 510640, China
[e.] MOE Key Laboratory of Laser Life Science and Institute of Laser Life Science, College of Biophotonics, South China Normal University, 55 Zhongshan Avenue West, Guangzhou 510631, China. E-mail: xlhu@scnu.edu.cn

‡ These authors contributed equally to this work.

Experimental section

Chemicals and Methods. All chemicals and reagents were commercially available and used as received without further purification. 9,10-Anthracenediyl-bis(methylene)dimalonic acid (ABDA), 2',7'-dichlorodihydrofluorescein diacetate (H2DCF-DA) and 3-(4,5-dimethyl-2-thiazolyl)-2,5-diphenyltetrazolium bromide (MTT) were purchased from Sigma-Aldrich and used as received. For cell culture, minimum essential medium (MEM), fetal bovine serum (FBS), penicillin-streptomycin solution, MitoTracker Green were purchased from Invitrogen. ^{1}H and ^{13}C NMR spectra were measured on a Bruker ARX 400 NMR spectrometer using $CDCl_3$ and DMSO-d_6 as solvents and tetramethylsilane (TMS; $\delta = 0$ ppm) was chosen as internal reference. High-resolution mass spectra (HR-MS) were obtained on a Finnigan MAT TSQ 7000 Mass Spectrometer System operated in a MALDI-TOF mode. Absorption spectra were measured on a Milton Roy Spectronic 3000 Array spectrophotometer. Steady-state photoluminescence (PL) spectra were measured on a Perkin-Elmer spectrofluorometer LS 55. Absolute fluorescence quantum yield was measured by a calibrated integrating sphere (Labsphere). Single crystal data was collected on a SuperNova, Dual, Cu at zero, Atlas diffractometer. The crystal was kept at 100.01(10) K during data collection. Using Olex2, the structure was solved with the Superflip structure solution program using Charge Flipping and refined with the ShelXL refinement package using Least Squares minimisation. Two-photon excitation fluorescence cross-section was measured by two-photon excitation fluorescence method using rhodamine B as reference. The excitation source for two-photon excitation was a femtosecond optical parametric amplifier (Coherent OPerA Solo) pumped by an amplified Ti:Sapphire system (Coherent Legend Elite system) and then detected with a spectrometer (Acton SpectraPro-500i) coupled to an CCD. Simulation was carried out with the Gaussian 09 package. Laser confocal scanning microscope images were collected on Zeiss laser scanning confocal

microscope (LSM 710) and analyzed using ZEN 2009 software (Carl Zeiss).

Synthesis of 2-bromo-9-ethyl-9H-carbazole. To a mixture of 2-bromo-9H-carbazole (2.27 g, 9.23 mmol) and anhydrous DMF (30 mL) was added slowly NaH (0.52 g, 60% w/w dispersion in mineral oil). After 30 min, bromoethane (0.89 mL, 11.92 mmol) was added and the solution was stirred for 18 h under argon. The reaction was quenched with water and extracted with dichloromethane. The organic fractions were dried over $MgSO_4$ and the solvent was removed under reduced pressure. The product was purified by gel column chromatography eluting with ethyl acetate/hexane (1:10, v:v) giving the title compound as a white solid (2.37 g, yield: 94%). ^{1}H NMR (400 MHz, $CDCl_3$, ppm): δ 8.08 (d, J = 7.8 Hz, 1H), 7.95 (d, J = 8.3 Hz, 1H), 7.57 (d, J = 1.6 Hz, 1H), 7.52-7.48 (m, 1H), 7.42 (d, J = 8.2 Hz, 1H), 7.35 (dd, J = 8.3, 1.7 Hz, 1H), 7.28-7.24 (m, 1H), 4.34 (q, J = 7.2 Hz, 2H), 1.45 (t, J = 7.2 Hz, 3H). ^{13}C NMR (100 MHz, $CDCl_3$, ppm): δ 140.15, 139.46, 125.49, 121.83, 121.32, 121.29, 120.95, 119.83, 118.76, 118.66, 110.96, 108.07, 37.02, 13.16. HRMS (MALDI-TOF): m/z calcd. for $C_{14}H_{12}BrN$ $[M]^+$: 273.0153, found: 273.0133.

Synthesis of 9-ethyl-9H-carbazole-2-carbaldehyde. A solution of 2-bromo-9-ethyl-9H-carbazole (2.20g, 8.04 mmol) in anhydrous THF (25 mL) was cooled to −78 °C under argon using an acetone dry-ice bath. n-BuLi (3.22 mL, 8.04 mmol, 2.5 M in hexane) was added dropwise over 30 min. Dry DMF (0.92 mL) was added slowly to the reaction solution after 1 h at −78 °C. After stirring for 2 h at −78 °C, the mixture was brought back to room temperature and the reaction was quenched by carefully adding HCl (2 N). The solution was extracted with CH_2Cl_2. The organic layer was dried over Na_2SO_4, filtred and concentrated. The crude product was purified by column chromatography on silica gel eluting with ethyl acetate/hexane (1:10, v:v) to afford a faint yellow solid (1.52 g, yield: 85%). ^{1}H NMR (400 MHz, $CDCl_3$, ppm): δ 10.17 (s, 1H), 8.22 (d, J = 7.9 Hz, 1H), 8.17 (d, J = 7.8 Hz, 1H), 7.98 (s, 1H), 7.75 (dd, J = 8.0, 1.2 Hz, 1H), 7.59-7.55 (m, 1H), 7.47

(d, J = 8.3 Hz, 1H), 7.31-7.27 (m, 1H), 4.44 (q, J = 7.2 Hz, 2H), 1.48 (t, J = 7.2 Hz, 3H). ^{13}C NMR (100 MHz, $CDCl_3$, ppm): δ 192.06, 141.08, 138.94, 133.19, 127.56, 127.01, 121.37, 120.90, 120.61, 119.96, 118.98, 108.79, 108.36, 37.11, 13.28. HRMS (MALDI-TOF): m/z calcd. for $C_{15}H_{13}NO$ $[M]^+$: 223.0997, found: 223.0998.

Synthesis of 7-bromo-9-ethyl-9H-carbazole-2-carbaldehyde. The same synthetic procedure as described for 9-ethyl-9H-carbazole-2-carbaldehyde using 2,7-dibromo-9-ethyl-9H-carbazole (2.84g, 8.04 mmol), affording the product as a white solid (2.0 g, yield: 82%). ^1H NMR (400 MHz, $CDCl_3$, ppm): δ 10.17 (s, 1H), 8.19 (d, J = 8.0 Hz, 1H), 8.00 (d, J = 8.3 Hz, 1H), 7.97 (s, 1H), 7.77 (dd, J = 8.0 Hz, 1.2 Hz, 1H), 7.62 (d, J = 1.5 Hz, 1H), 7.40 (dd, J = 8.3 Hz, 1.6 Hz, 1H), 4.41 (q, J = 7.2 Hz, 2H), 1.48 (t, J = 7.3 Hz, 3H).

Synthesis of 7-(diphenylamino)-9-ethyl-9H-carbazole-2-carbaldehyde. 7-bromo-9-ethyl-9*H*-carbazole-2-carbaldehyde (0.31 g, 1.02 mmol), diphenylamine (0.26 g, 1.54 mmol) and Cs_2CO_3 (0.50 g, 1.54 mmol) were mixed in toluene (5 mL) under argon. Then $Pd(OAc)_2$ (0.012 g, 0.054 mmol) and $P(tBu)_3$ (26 μL, 0.11 mmol.) were added in the mixture. The final solution was heated at 120 °C for 24 h. After cooling back to room temperature, the reaction mixture was washed with water, 1 M HCl and water again, dried over Na_2SO_4 and concentrated. The crude product was purified by chromatography on silica gel eluting with ethyl acetate/hexane (1:10, v:v) giving 7-(diphenylamino)-9-ethyl-9H-carbazole-2-carbaldehyde as yellow solid (0.35 g, yield: 88%). ^1H NMR (400 MHz, $CDCl_3$, ppm): δ 10.12 (s, 1H), 8.09 (d, J = 8.0 Hz, 1H), 7.97 (d, J = 8.5 Hz, 1H), 7.90 (s, 1H), 7.72 (dd, J = 8.0 Hz, 1.2 Hz, 1H), 7.31-7.27 (m, 4H), 7.18-7.16 (m, 4H), 7.10-7.04 (m, 3H), 7.01 (dd, J = 8.5 Hz, 1.8 Hz, 1H), 4.26 (q, J = 7.2 Hz, 2H), 1.35 (t, J = 7.2 Hz, 3H).

Synthesis of N-(2-hydroxyethyl)-4-methylpyridinium bromide. A solution of 4-picoline (1.27 g, 13.60 mmol) and 2-bromoethanol (1.70 g, 13.60 mmol) in acetonitrile (6 mL) was refluxed

for 3 h and then cooled to room temperature. Then the viscous mixture was washed by ethyl ether (30 mL) for three times and dried under vacuum, affording product as viscous oil (0.87 g, yield: 95%). ^{1}H NMR (400 MHz, DMSO-d_6, ppm): 8.87 (d, J = 6.5 Hz, 2H), 7.98 (d, J = 6.3 Hz, 2H), 5.20 (s, 1H), 4.60 (t, J = 4.94 Hz, 2H), 3.81 (t, J = 4.94 Hz, 2H), 2.59 (s, 3H). ^{13}C NMR (100 MHz, DMSO-d_6, ppm): δ 158.61, 143.86, 127.75, 61.79, 59.78, 21.30.

Synthesis of N-(2-hydroxyethyl)-4-methylquinolinium bromide. The same synthetic procedure as described for N-(2-hydroxyethyl)-4-methylpyridinium bromide using lepidine (1.95g, 13.60 mmol) and 2-bromoethanol (1.70 g, 13.60 mmol), affording product as gray solid (2.37 g, yield: 65%). ^{1}H NMR (400 MHz, DMSO-d_6, ppm): 9.22 (d, J = 6.1 Hz, 1H), 8.59 (d, J = 8.9 Hz, 1H), 8.53 (d, J = 8.5 Hz, 1H), 8.27-8.17 (m, 1H), 8.09-7.98 (m, 2H), 5.14 (t, J = 5.6 Hz, 1H), 5.06 (t, J = 4.9 Hz, 2H), 3.89 (q, J = 5.1 Hz, 2H), 2.99 (s, 3H).

Synthesis of CPy. 9-ethyl-9H-carbazole-2-carbaldehyde (0.28 g, 1.28 mmol) and N-(2-hydroxyethyl)-4-methylpyridinium bromide (0.25 g, 1.16 mmol) were dissolved in dry ethanol (15 mL). 2 drops of piperidine was added and the solution was refluxed for 3 h under nitrogen. After cooling to room temperature, the precipitated solid was filtered, washed with cold ethanol and dried to give the bromide salt of the product as a yellow solid. Then the solid was dissolved in acetone (20 mL) and a saturated aqueous solution of NH_4PF_6 (20 mL) was then added. After stirring for 30 min, the solution was evaporated to dryness. The residue was purified by flash silica gel column chromatography eluting with dichloromethane/methanol (20:1, v:v) giving CPy as yellow solid (0.37 g, yield: 65%). ^{1}H NMR (400 MHz, DMSO-d_6, ppm): δ 8.86 (d, J = 6.4 Hz, 2H), 8.26-8.18 (m, 5H), 8.02 (s, 1H), 7.69-7.61 (m, 3H), 7.51 (t, J = 7.5 Hz, 1H), 7.23 (t, J = 7.4 Hz, 1H), 5.27 (s, 1H), 4.54-4.49 (m, 4H), 3.86 (s, 2H), 1.37 (t, J = 7.0 Hz, 3H). ^{13}C NMR (100 MHz, DMSO-d_6, ppm): δ 153.00, 144.40, 141.99, 140.49, 139.50, 132.47, 126.55, 123.93, 122.96, 122.11, 121.60,

120.69, 119.04, 109.18, 108.89, 61.89, 59.86, 36.88, 13.60. HRMS (MALDI-TOF): m/z calcd. for $C_{23}H_{23}N_2O$ [M-PF$_6$]$^+$: 343.1805, found: 343.1806.

Synthesis of CQu. The same synthetic procedure as described for CPy using 9-ethyl-9H-carbazole-2-carbaldehyde (0.28 g, 1.28 mmol) and N-(2-hydroxyethyl)-4-methylquinolinium bromide (0.31 g, 1.16 mmol), affording CQu as red solid (0.38 g, yield: 60%). ^{1}H NMR (400 MHz, DMSO-*d*$_6$, ppm): 9.24 (d, *J* = 6.4 Hz, 1H), 9.13 (d, *J* = 8.5 Hz, 1H), 8.58-8.47 (m, 3H), 8.38 (d, *J* = 15.8 Hz, 1H), 8.30-8.20 (m, 4H), 8.08 (t, *J* = 7.6 Hz, 1H), 7.86 (d, *J* = 8.1 Hz, 1H), 7.66 (d, *J* = 8.2 Hz, 1H), 7.52 (t, *J* = 7.5 Hz, 1H), 7.24 (t, *J* = 7.4 Hz, 1H), 5.19 (s, 1H), 5.05 (s, 2H), 4.57 (q, 2H), 3.94 (s, 2H), 1.40 (t, *J* = 6.8 Hz, 3H). ^{13}C NMR (100 MHz, DMSO-*d*$_6$, ppm): δ 152.85, 147.89, 144.40, 140.58, 139.56, 137.92, 134.67, 132.83, 128.82, 126.65, 126.56, 126.52, 124.21, 121.59, 120.76, 120.64, 120.13, 119.08, 118.55, 115.36, 109.34, 109.22, 58.78, 58.64, 36.91, 13.69. HRMS (MALDI-TOF): m/z calcd. for $C_{27}H_{25}N_2O$ [M-PF$_6$]$^+$: 393.1961, found: 393.1932.

Synthesis of DCPy. The same synthetic procedure as described for CPy using 7-(diphenylamino)-9-ethyl-9H-carbazole-2-carbaldehyde (0.5 g, 1.28 mmol) and N-(2-hydroxyethyl)-4-methylpyridinium bromide (0.25 g, 1.16 mmol), affording DCPy as dark red solid (0.66 g, yield: 87%). ^{1}H NMR (400 MHz, DMSO-*d*$_6$, ppm): 8.84 (d, *J* = 6.5 Hz, 2H), 8.22-8.13 (m, 4H), 8.08 (d, *J* = 8.4 Hz, 1H), 7.96 (s, 1H), 7.66-7.58 (m, 2H), 7.33-7.29 (m, 4H), 7.19 (s, 1H), 7.07-7.03 (m, 6H), 6.87 (d, *J* = 8.5 Hz, 1H), 5.29 (s, 1H), 4.53 (s, 2H), 4.57 (q, *J* = 6.7 Hz, 2H), 3.86 (s, 2H), 1.25 (t, *J* = 7.0 Hz, 3H). ^{13}C NMR (100 MHz, DMSO-*d*$_6$, ppm): δ 153.07, 147.36, 146.51, 144.39, 142.13, 141.61, 140.01, 131.76, 129.33, 124.14, 123.52, 122.91, 122.74, 121.81, 121.73, 120.09, 119.57, 117.62, 116.77, 108.63, 104.17, 61.86, 59.87, 36.80, 13.46. HRMS (MALDI-TOF): m/z calcd. for $C_{35}H_{32}N_3O$ [M-PF$_6$]$^+$: 510.2540, found: 510.2569.

Synthesis of DCQu. The same synthetic procedure as described for CPy using 7-(diphenylamino)-9-ethyl-9H-carbazole-2-carbaldehyde (0.5 g, 1.28 mmol) and 1-(2-hydroxyethyl)-4-methylquinolinium iodide (0.37 g, 1.16 mmol), affording DCQu as purple crystalline solid (0.63 g, yield: 77%). ^{1}H NMR (400 MHz, DMSO-d_6, ppm): δ 9.20 (d, J = 6.6 Hz, 1H), 9.11 (d, J = 8.4 Hz, 1H), 8.55 (d, J = 9.0 Hz, 1H), 8.51 (d, J = 6.6 Hz, 1H), 8.45 (d, J = 15.9 Hz, 1H), 8.35 (d, J = 15.8 Hz, 1H), 8.25-8.22 (m, 2H), 8.17 (d, J = 8.1 Hz, 1H), 8.10-8.03 (m, 2H), 7.81 (d, J = 7.8 Hz, 1H), 7.33-7.29 (m, 4H), 7.18 (s, 1H), 7.08-7.03 (m, 6H), 6.87 (dd, J = 8.4 Hz, 1.7 Hz, 1H), 5.19 (t, J = 5.6 Hz, 1H), 5.04 (t, J = 4.5 Hz, 2H), 4.36 (q, J = 6.2 Hz, 2H), 3.93 (q, J = 4.8 Hz, 2H), 1.27 (t, J = 7.1 Hz, 3H). ^{13}C NMR (100 MHz, DMSO-d_6, ppm): δ 152.96, 147.91, 147.35, 146.67, 144.61, 141.77, 140.12, 137.99, 134.70, 132.12, 129.36, 128.83, 126.59, 126.54, 124.51, 123. 63, 122.83, 121.86, 120.76, 120.32, 120.02, 118.22, 117.59, 116.68, 115.30, 109.03, 104.03, 58.79, 58.64, 36.84, 13.55. HRMS (MALDI-TOF): m/z calcd. for $C_{39}H_{34}N_3O$ $[M\text{-}PF_6]^+$: 560.2696, found: 560.2714.

Cell Lines. HeLa: human cervical cancer cell line; A549: adenocarcinomic human alveolar basal epithelial; HepG2: human liver cancer cell line; B16: murine melanoma tumor cell line; HLF: human lung fibroblast cell line; LX2: human hepatic stellate cell line.

Cell Culture. Cell lines were cultured in MEM containing 10% FBS and antibiotics (100 units per mL penicillin and 100 μg/mL streptomycin) in a 5% CO_2 humidity incubator at 37 °C.

Cell Imaging. Cells were grown in a 35-mm petri dish with a cover slip. The cells were stained with certain dye at certain concentration (by adding 2 μL of stock solution in DMSO to a 2 mL of MEM with DMSO < 0.1 vol %) for 30 min. For co-staining with MitoTracker Green, cells were firstly incubated with each AIEgens (CPy, CQu, DCPy and DCQu) and MitoTracker Green (0.5 μM) at 37 ˚C for 30 min. After incubation with dye, the cells were washed with PBS for three times.

The cells were imaged under a confocal microscope (Zeiss LSM 710 Laser Scanning Confocal Microscope), using proper excitation and emission filters for each dye: for AIEgens, excitation filter = 488 nm and emission filer = 600−740 nm; for MitoTracker Green, excitation filter = 488 nm and emission filter = 500−530 nm. The mitochondrial disruption process monitoring was performed by continuous scanning for 15 min.

Photostability. The HeLa Cells labelled with certain dyes were imaged by a confocal microscope (Zeiss LSM 710 Laser Scanning Confocal Microscope), the dyes were excited with 488 nm laser light for one-photon imaging. Imaging parameters were set for each dye individually to obtain optimal images. Continues scans (11s per Scan) were taken. On each series of scans, three regions of interest (ROIs) with mitochondria were defined. The first scan of each ROI was set to 100%. Then the pixel intensity values for each ROI were averaged and plotted against the scan number. The resulting curve represented the bleaching rate that an experimentalist would encounter.

Cytotoxicity of DCQu to cells under light irradiation. Cytotoxicity was evaluated by the 3-(4,5-Dimethythiazol-2-yl)-2,5-diphenyltetrazolium bromide (MTT) assays in accordance to the manufacturer manual. Cells were seeded in 96-well plates (Costar, IL, USA) at a density of 6000–8000 cells per well. After overnight culturing, medium in each well was replaced by 100 μL fresh medium containing different concentrations of DCQu or Ce6. The volume fraction of DMSO is below 0.2%. After incubation for 30 min, plates containing cells with fresh medium were exposed to white light (4.2 mW/cm^{-2}) for 90 min and another array of plates with cells was kept in dark as control. Then, the plates were conducted the same treatment as the biocompatibility test. After 24 h, 10 μL of MTT solution (5 mg/mL in PBS) was added into each well. After 4 h incubation, DMSO was added into each well and the plate was gently shaken to dissolve all the precipitated formed.

Finally, the absorption of each well at 570 nm was recorded *via* a plate reader (Perkin-Elmer Victor3TM). Each trial was performed with five wells parallel.

Cell apoptosis study. HeLa cells were stained with DCQu for 30 mins, washed with PBS for 3 times and irradiated with white light for 30 min. Then, cells were further stained with Annexin V-FITC for 15 min and imaged, excitation filter = 488 nm and emission filer = 500–600 nm. Cells with DCQu and Annexin V-FITC staining but without light irradiation were taken as control group.

Two-photon fluorescence imaging in cells. HeLa cells used for two-photon microscopy were stained with DCQu (5 μM) refer to the procedure described for confocal fluorescence imaging. Two-photon fluorescence images of HeLa cells were collected using a Stimulated emission depletion (STED) microscopy (Leica Stimulated Emission Depletion Microscope) equipped with a multiphoton laser (Coherent Chameleon Ultra II Multiphoton laser). Excitation wavelength = 900 nm; emission filter = 600–740 nm.

Two-photon PDT. HeLa cells were seeded 2×10^5 / dish in confocal image dish with 2 mL MEM medium supplied with 10% FBS and 1% PLS. After 24 hours, cells were stained with 5 μM DCQu for 30 min at 37 ℃, and then kept with fresh medium. Cells were then imaged under STED microscope equipped with two-photon laser with 900 nm excitation, 2500 W (67% gain). And images were taken after 1, 2, 4, 8, 16 and 32 scans.

$^1O_2$ generation detection. ABDA was used as the $^1O_2$-monitoring agent. In the experiments, 13 μL of ABDA stock solution (7.5 mM) was added to 2 mL of each AIEgen (CPy, CQu, DCPy and DCQu) suspension (5 μM) and white light (4.2 mW/cm^{-2}) was employed as the irradiation source. The absorption of ABDA at 378 nm was recorded at various irradiation time to obtain the decay rate of the photosensitizing process.

ROS generation detection by H2DCF-DA in cells. The HeLa cells were seeded in 35 mm petri

dish with cover slip at a density of 100 000 cells and incubated for 24 h. Cells were pre-incubated with 10 μM H2DCF-DA for 4 hours followed by staining with or without 5 μM DCQu for 30 min and then washed with 1×PBS for 3 times. The cells were carefully made into imaging slides and the slide was subjected to white light irradiation of maximum white light of CLSM 810 for 0, 0.5, 1.0 and 5 min followed by CLSM imaging (excitation: 488 nm, emission: 500-530 nm).

***In vivo* photodynamic therapy of melanoma.** Mouse B16 melanoma cells were cultured in 75T culture flasks in culture medium supplemented with 10% FBS at 37 ^{o}C in a 5% CO_2 incubator. Female C57BL/6 mice (6-7 weeks) were inoculated with B16 cells (2 x 10^6 cells/mL) on the rear dorsal area of each mouse. After ~7 days, the tumor reached a volume of ~50 mm^3 and used for followed therapy experiments. The tumor-bearing mice were randomly divided into 6 groups, including (1) PBS, (2) Light irradiation for 10 min, (3) Ce6 (10 μM) at dark, (4) DCQu (10 μM) at dark, (5) Ce6 with light irradiation, and (6) DCQu with light irradiation, respectively. The light irradiation was performed using a LED light with ultralow power density at 4.2 mW cm^{-2}. Typical intratumoral treatments were performed every three days and the mice photographs were recorded. Tumor growth was measured by measuring the tumor diameter with a caliper. The tumor volume was calculated using the following equation: $V = a * b^2/2$, where a and b are the largest and smallest diameters of the tumor. After diverse treatments, tumor tissues and major organs were harvested for histological analysis.

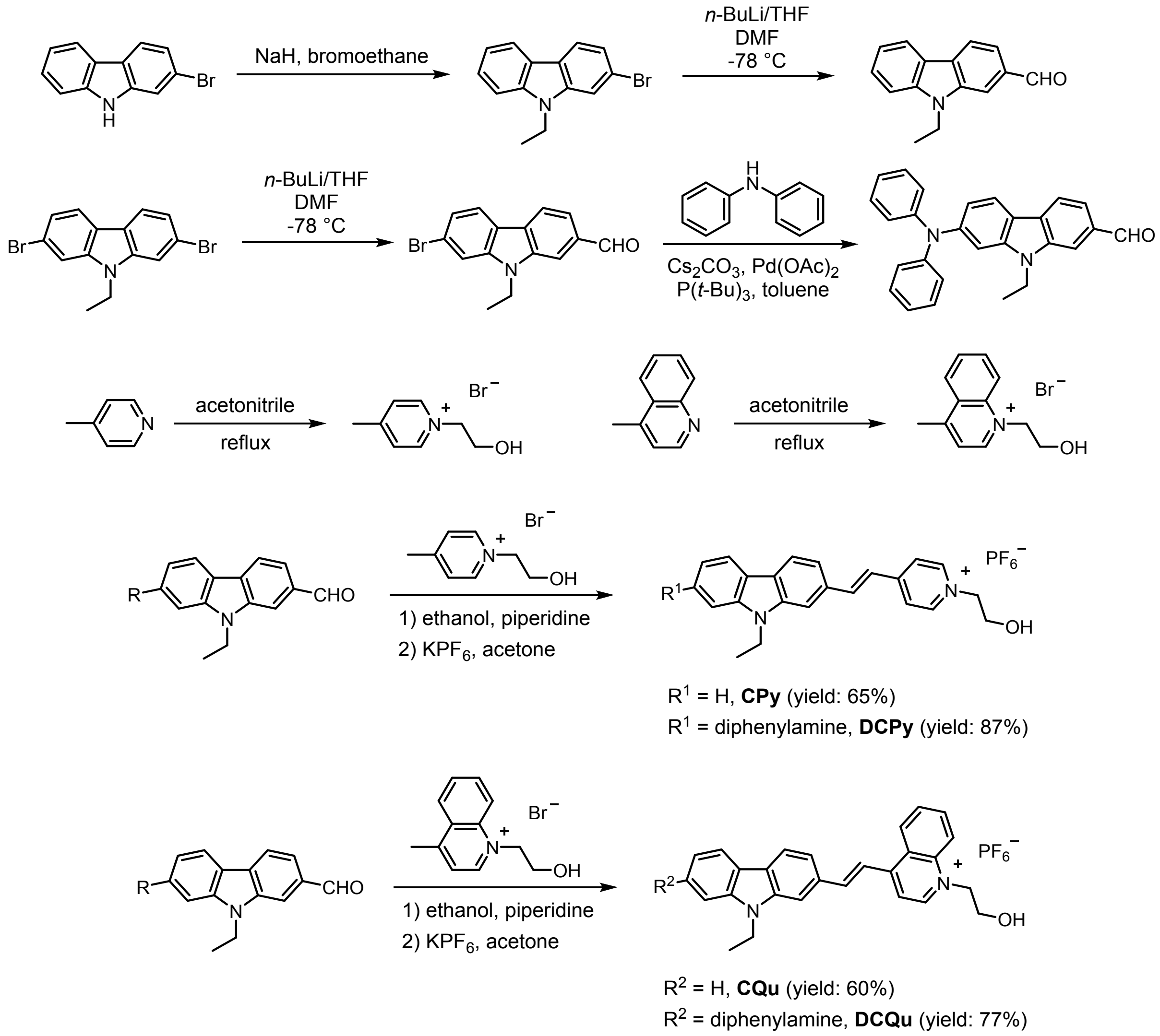

Sc

heme S1. Synthetic routes for the intermediates and AIEgens (CPy, CQu, DCPy and DCQu).

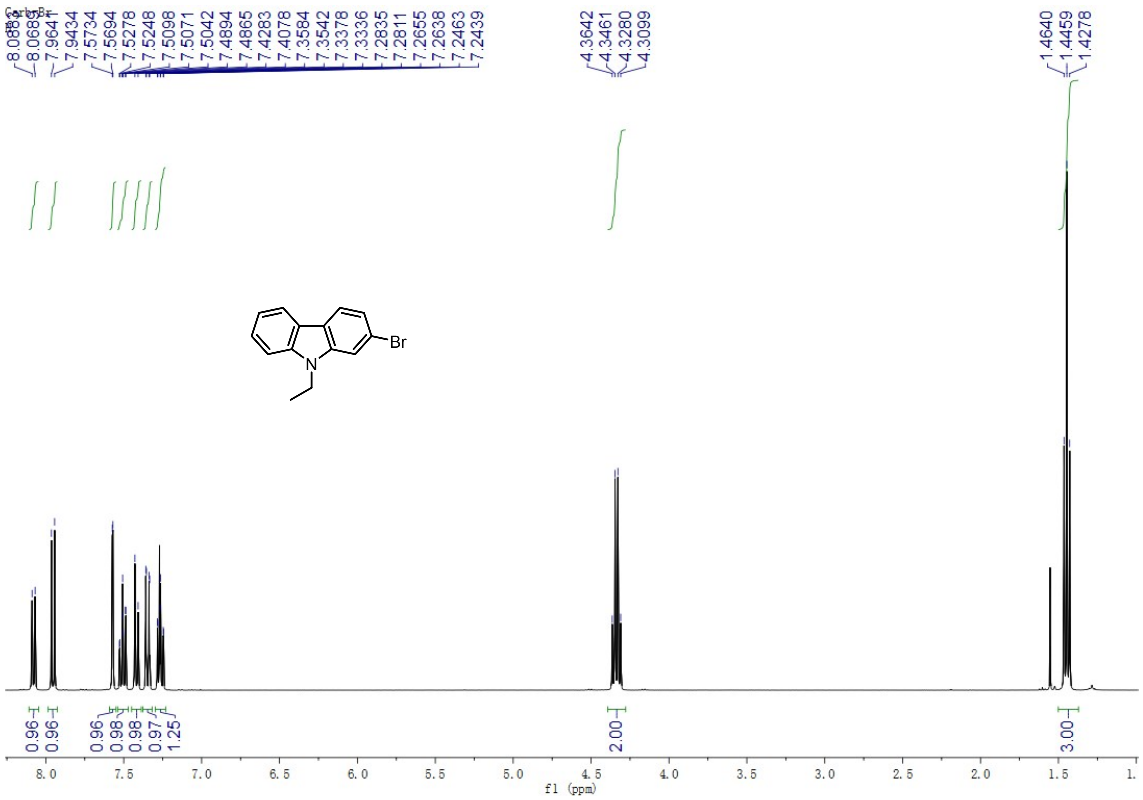

Figure S1. ^{1}H NMR spectrum of 2-bromo-9-ethyl-9H-carbazole in $CDCl_3$.

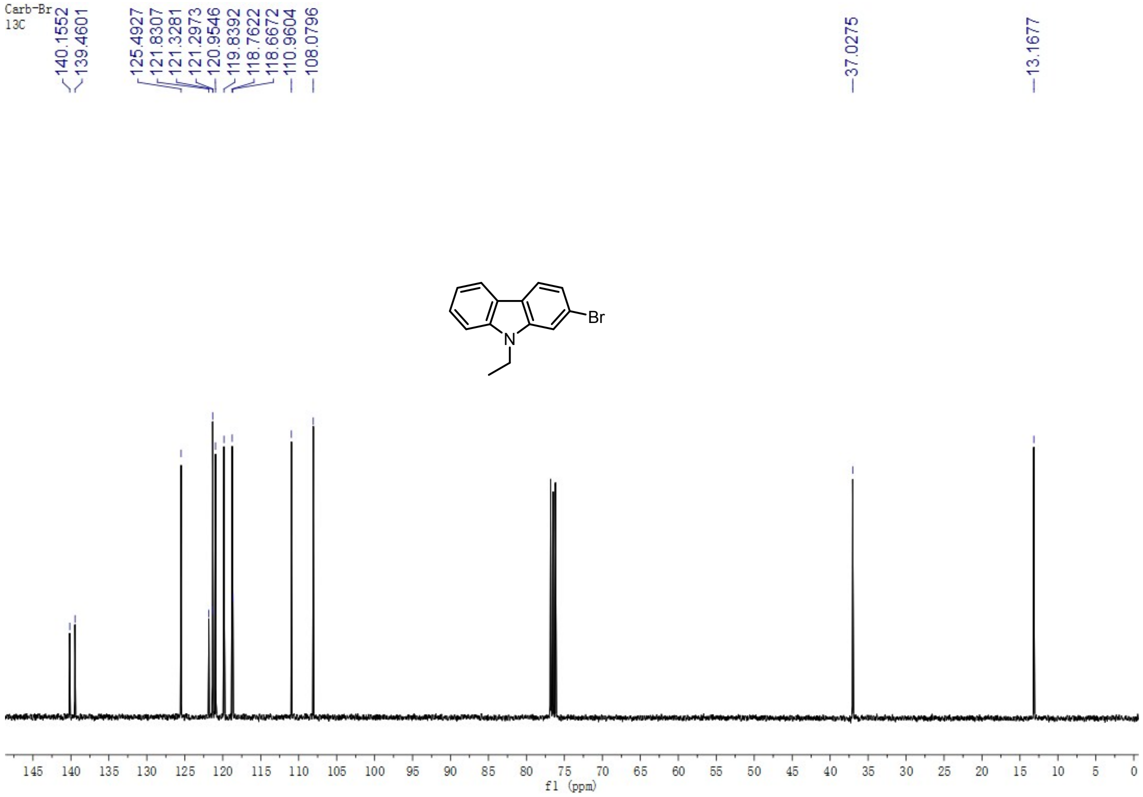

Figure S2. ^{13}C NMR spectrum of 2-bromo-9-ethyl-9H-carbazole in $CDCl_3$.

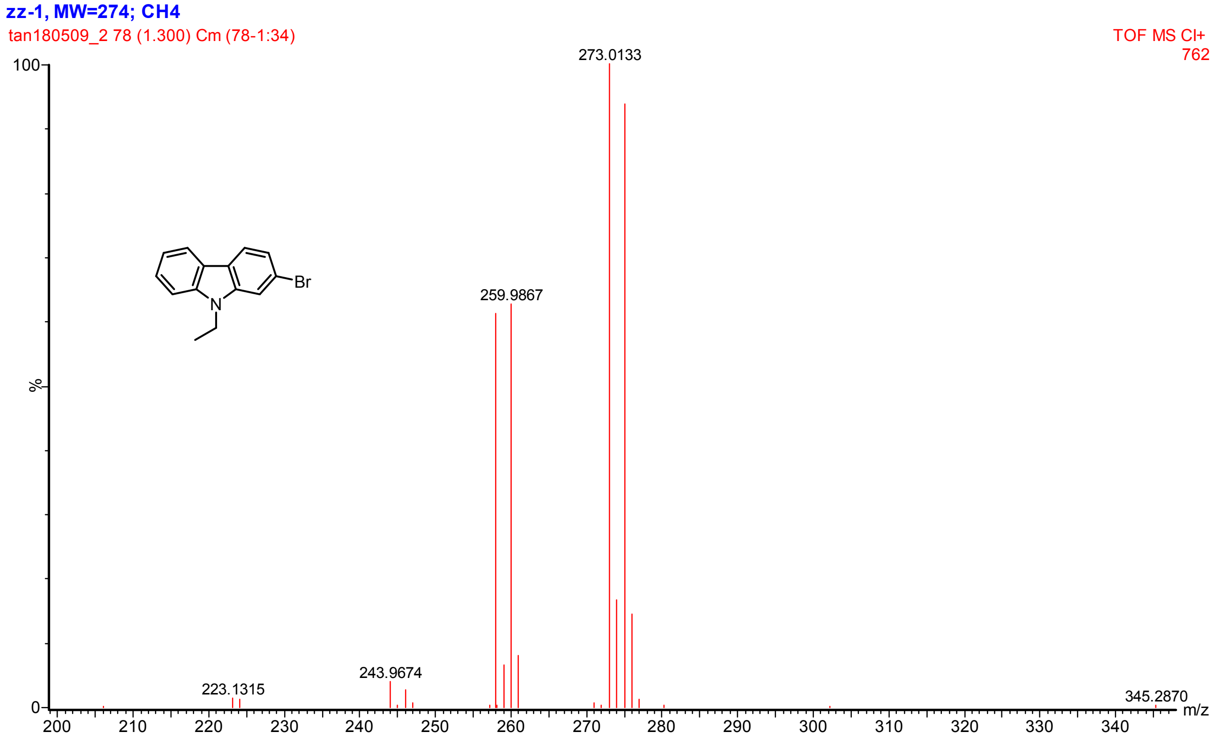

Figure S3. HRMS spectrum of 2-bromo-9-ethyl-9H-carbazole.

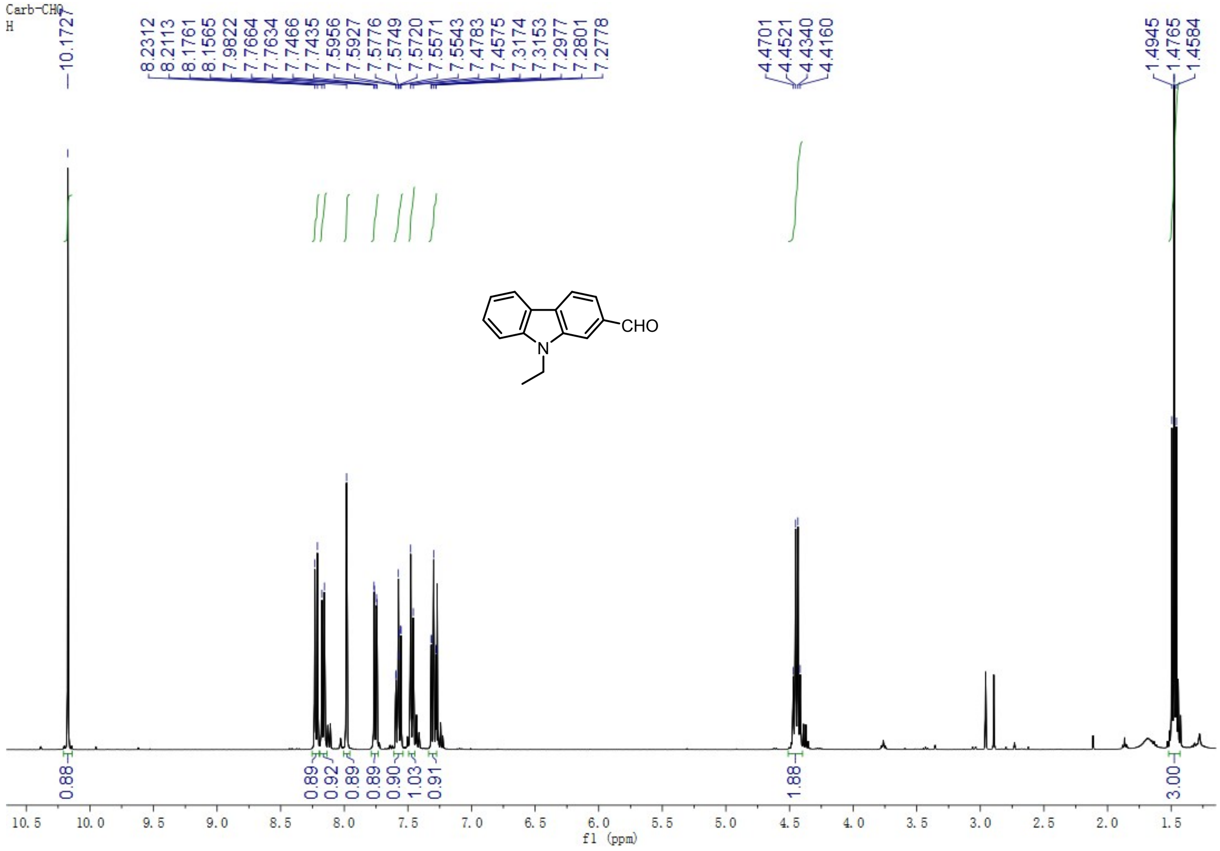

Figure S4. ^{1}H NMR spectrum of 9-ethyl-9H-carbazole-2-carbaldehyde in $CDCl_3$.

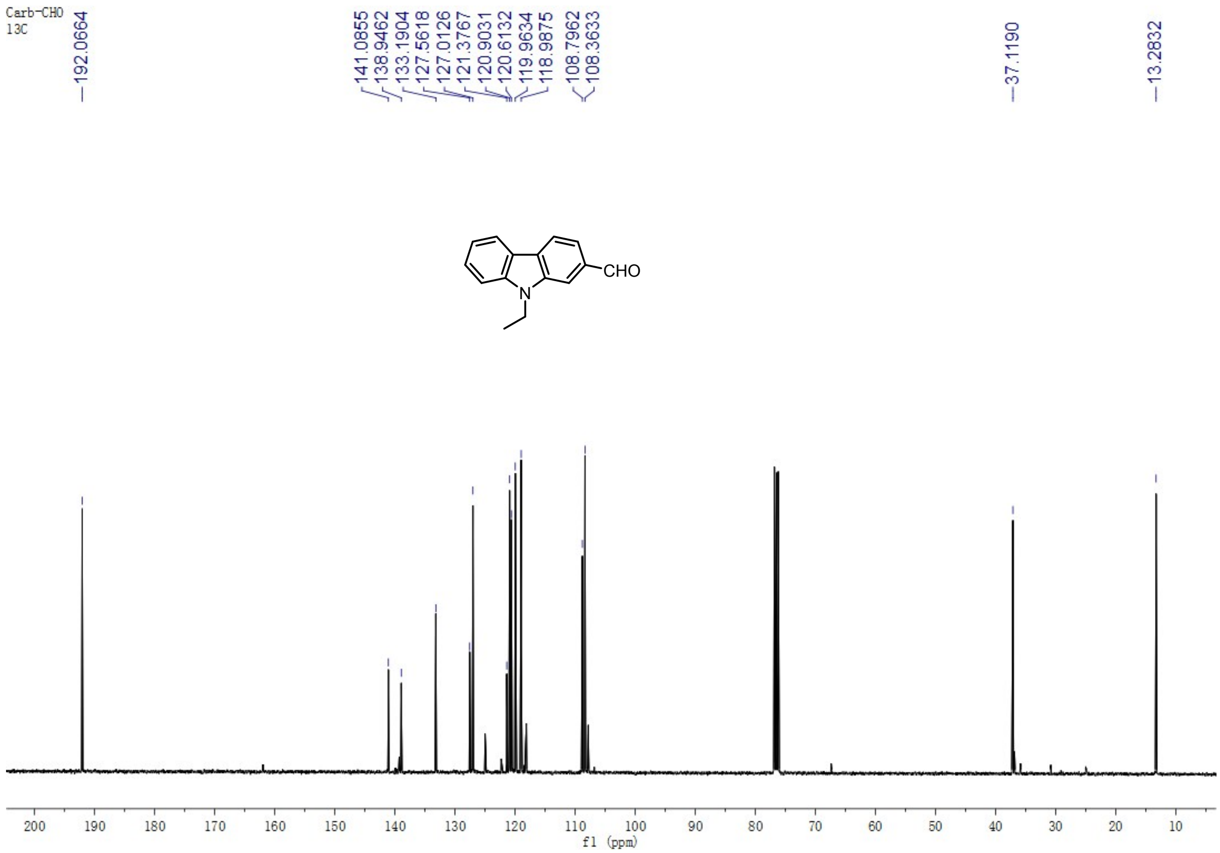

Figure S5. ^{13}C NMR spectrum of 9-ethyl-9H-carbazole-2-carbaldehyde in $CDCl_3$.

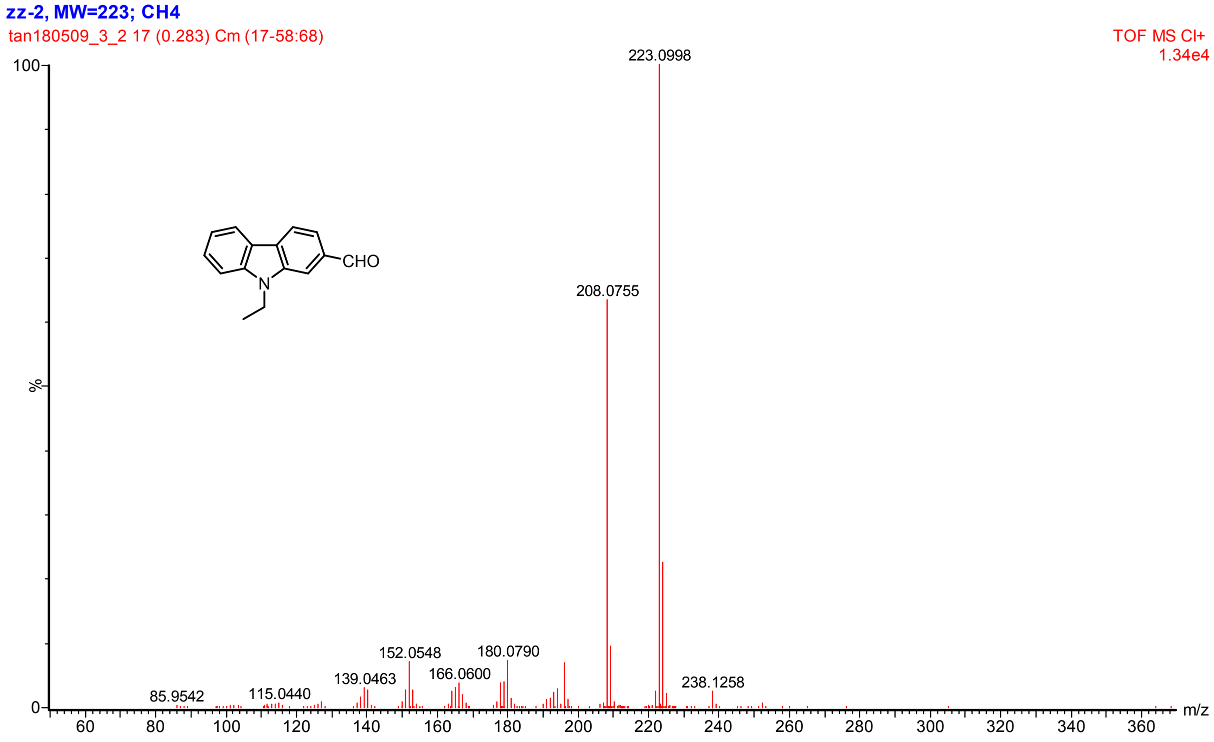

Figure S6. HRMS spectrum of 9-ethyl-9H-carbazole-2-carbaldehyde.

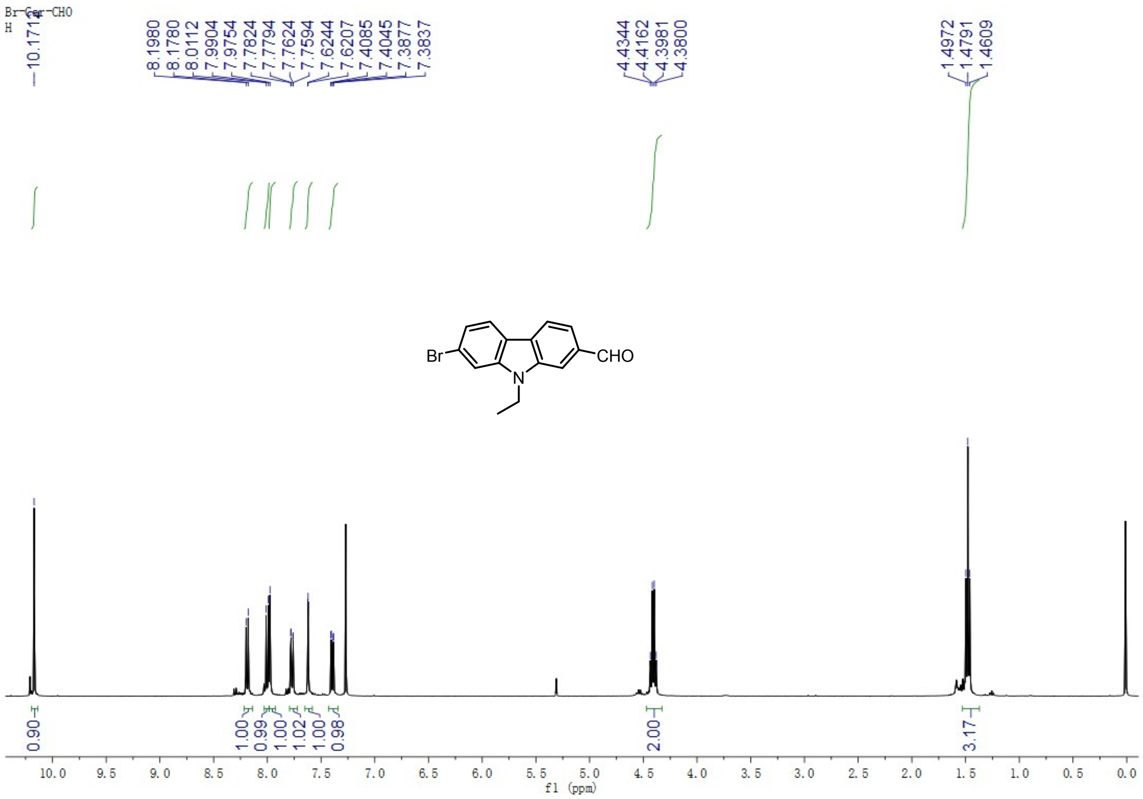

Figure S7. ^{1}H NMR spectrum of 7-bromo-9-ethyl-9*H*-carbazole-2-carbaldehyde in $CDCl_3$.

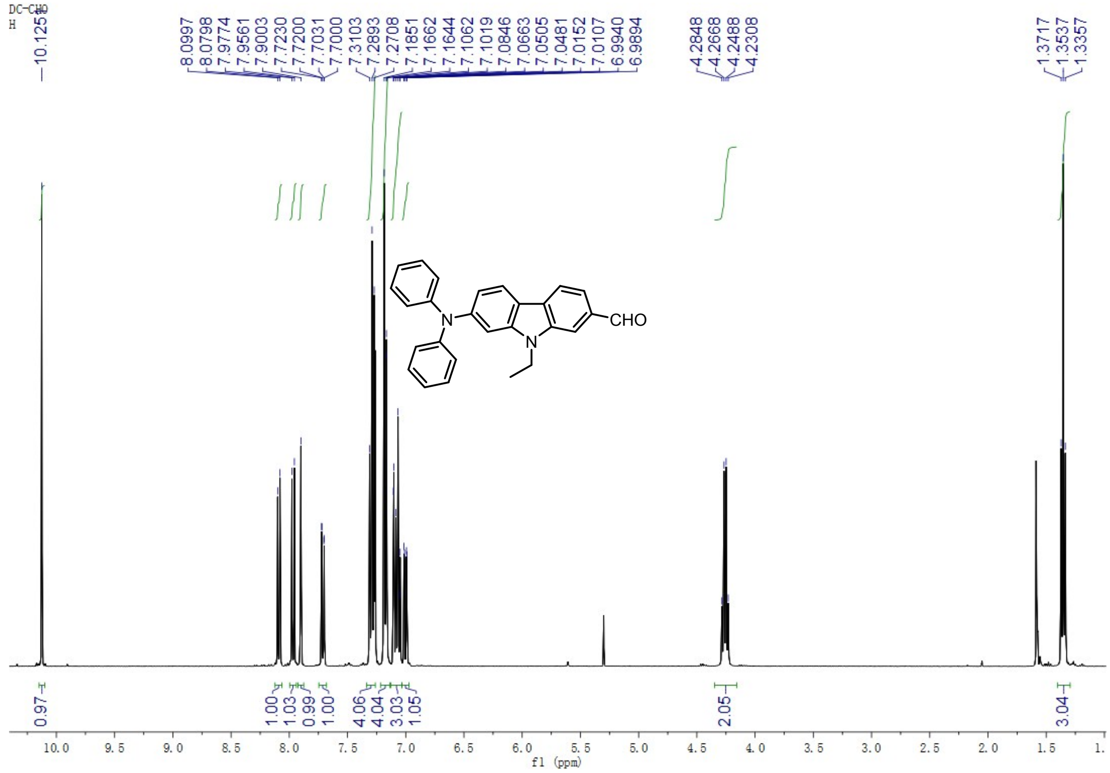

Figure S8. ^{1}H NMR spectrum of 7-(diphenylamino)-9-ethyl-9H-carbazole-2-carbaldehyde in $CDCl_3$.

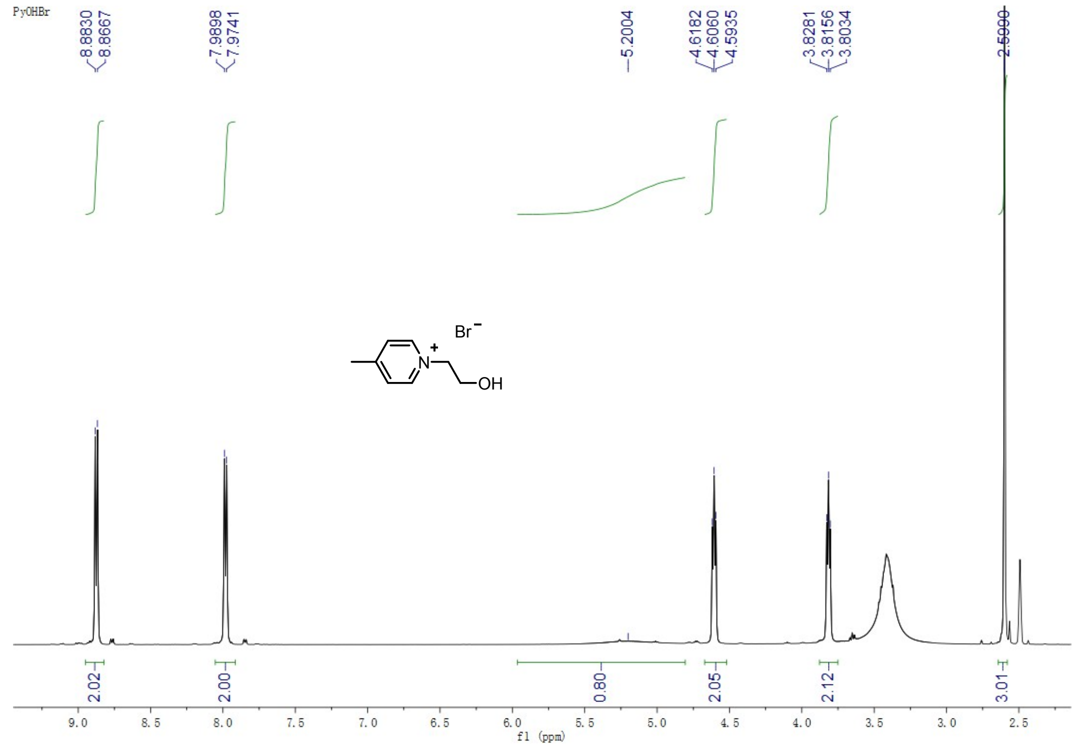

Figure S9. ^{1}H NMR spectrum of N-(2-hydroxyethyl)-4-methylpyridinium bromide (**3**) in DMSO-*d*$_6$.

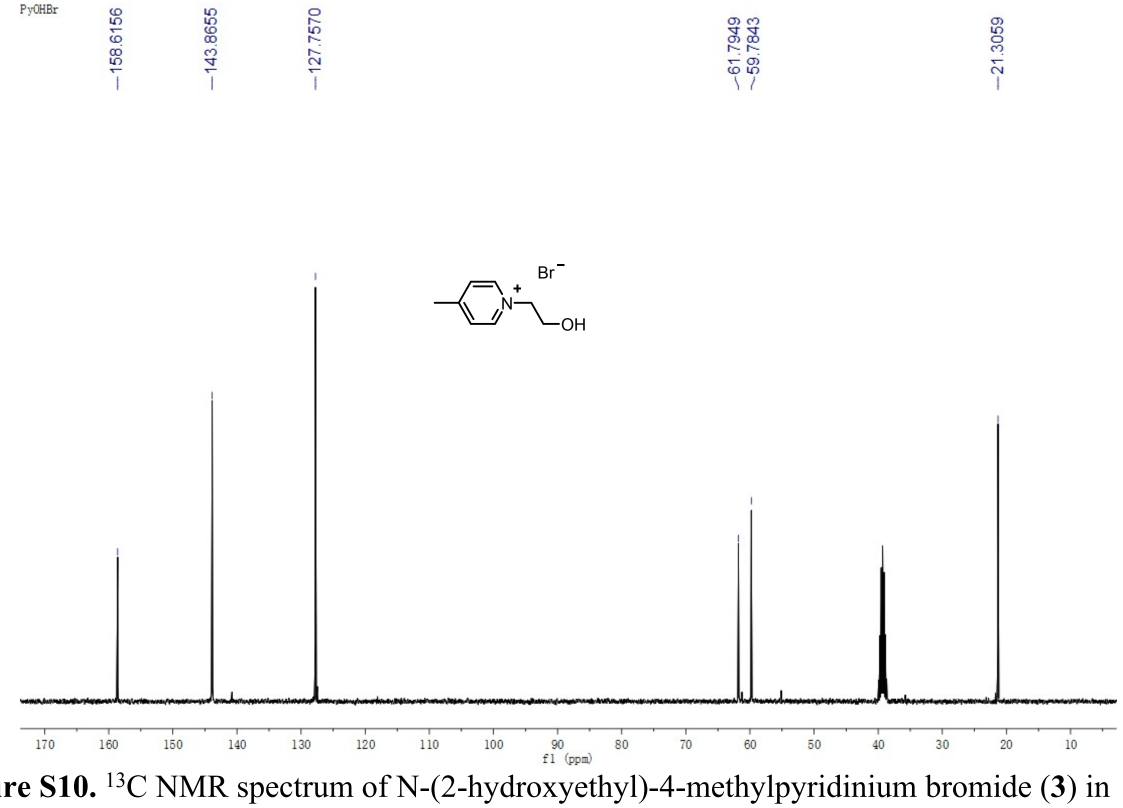

Figure S10. ^{13}C NMR spectrum of N-(2-hydroxyethyl)-4-methylpyridinium bromide (**3**) in DMSO-*d*$_6$.

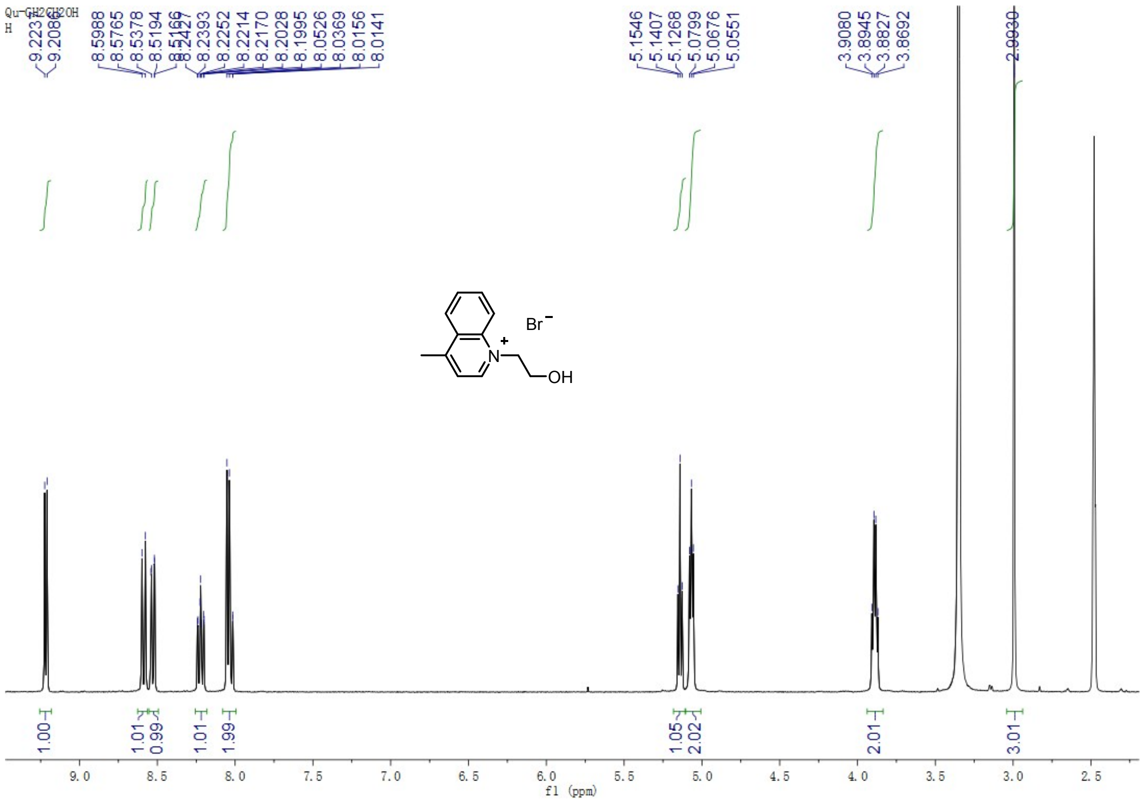

Figure S11. ^{1}H NMR spectrum of 1-(2-hydroxyethyl)-4-methylquinolinium iodide in DMSO-d_6.

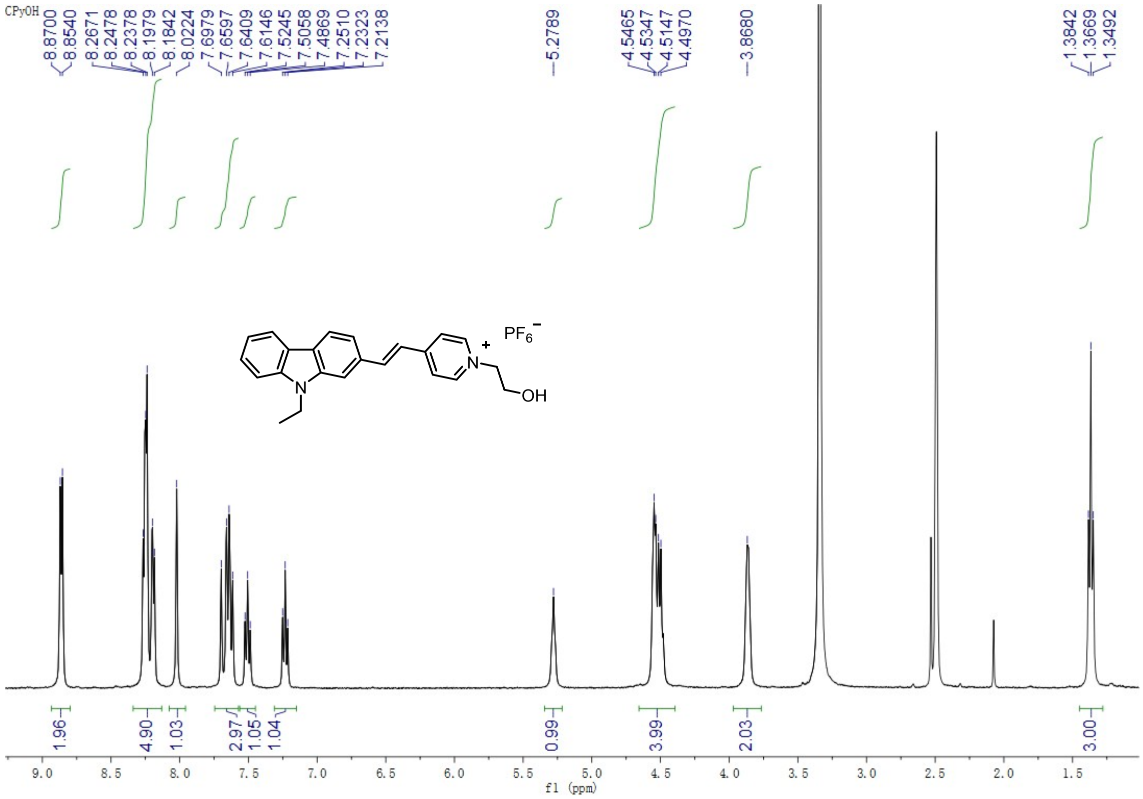

Figure S12. ^{1}H NMR spectrum of CPy in DMSO-d_6.

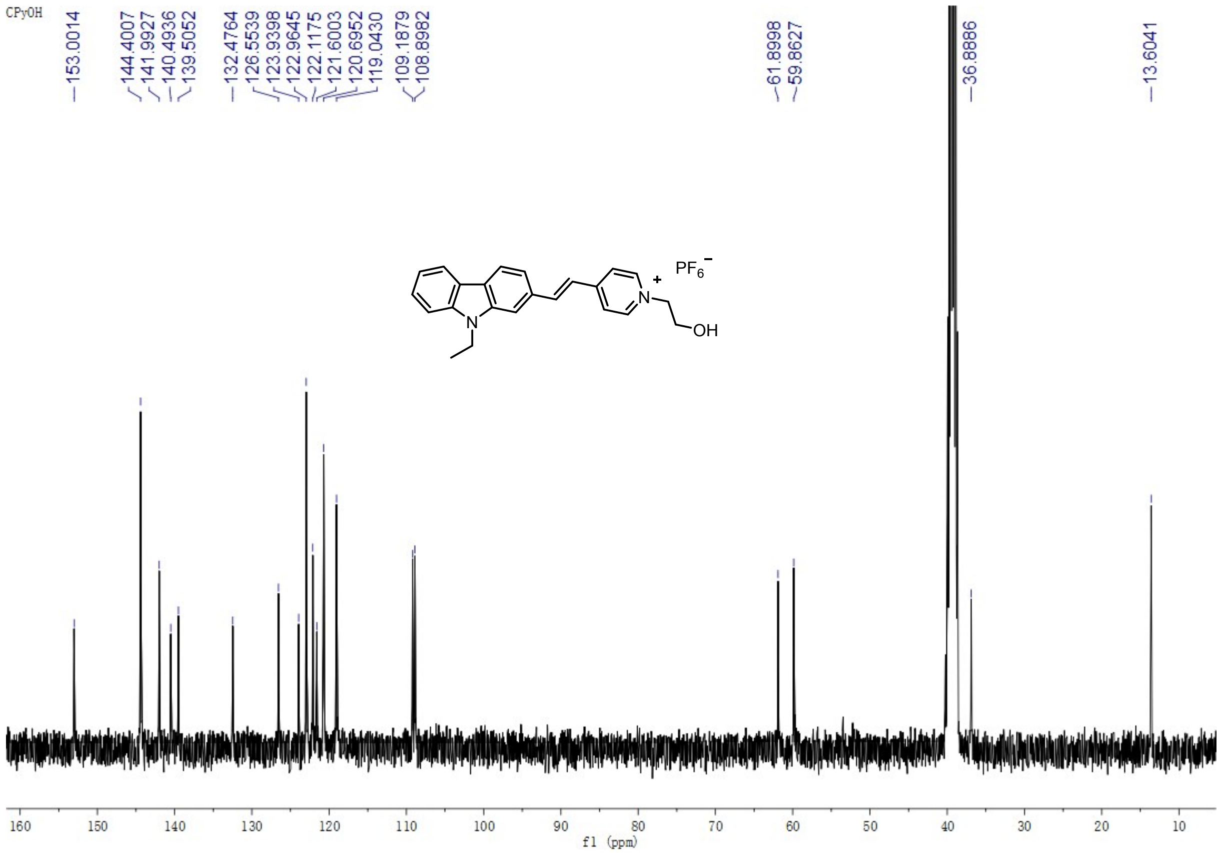

Figure S13. ^{13}C NMR spectrum of CPy in DMSO-d_6.

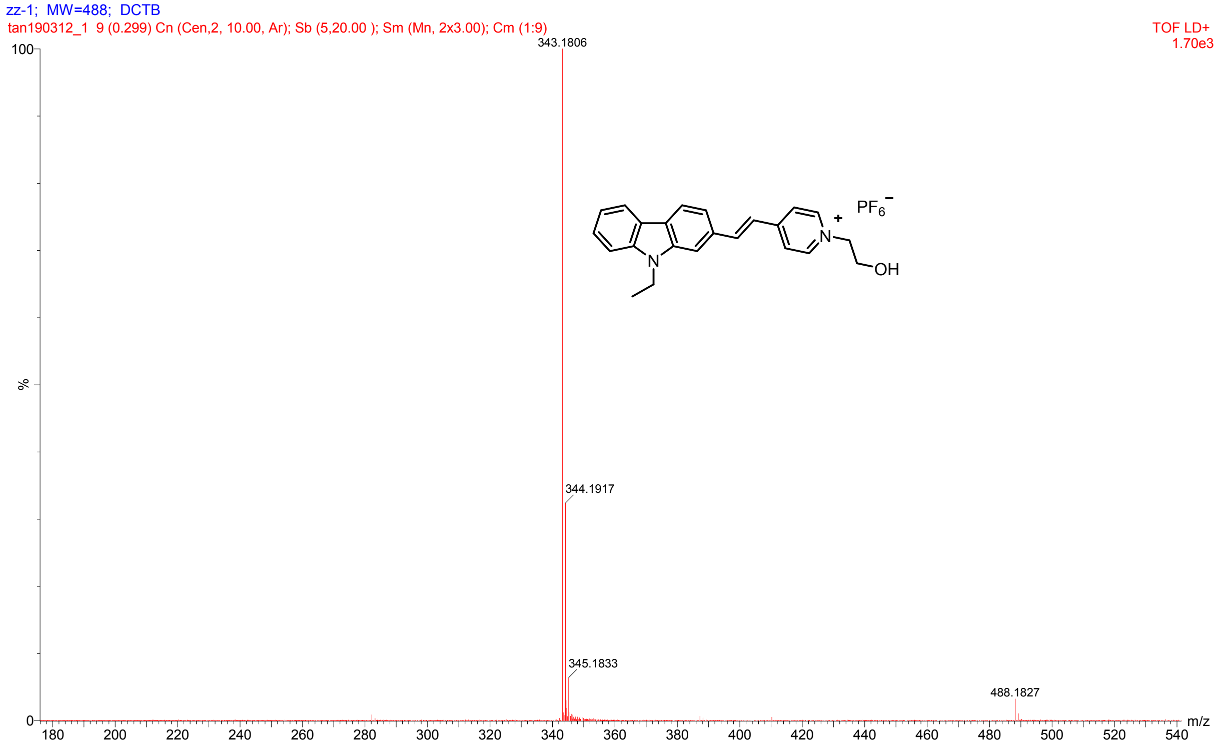

Figure S14. HRMS spectrum of CPy.

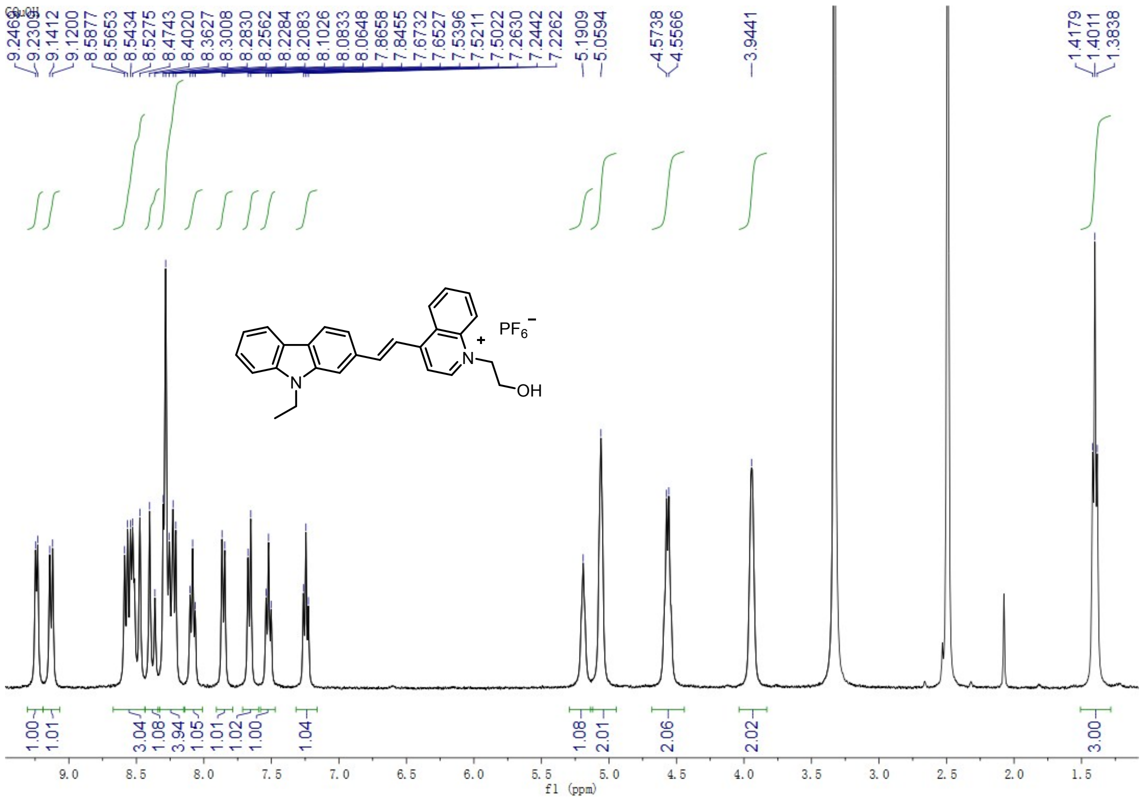

Figure S15. ^{1}H NMR spectrum of CQu in DMSO-d_6.

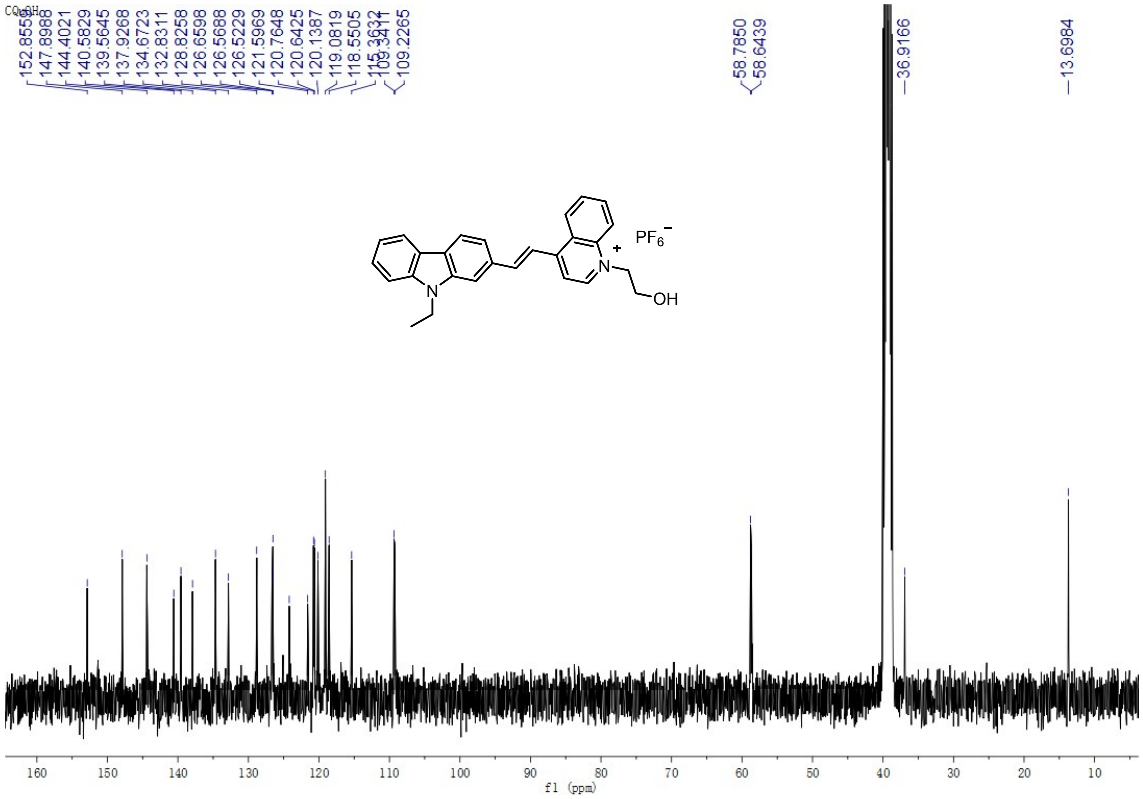

Figure S16. ^{13}C NMR spectrum of CQu in DMSO-d_6.

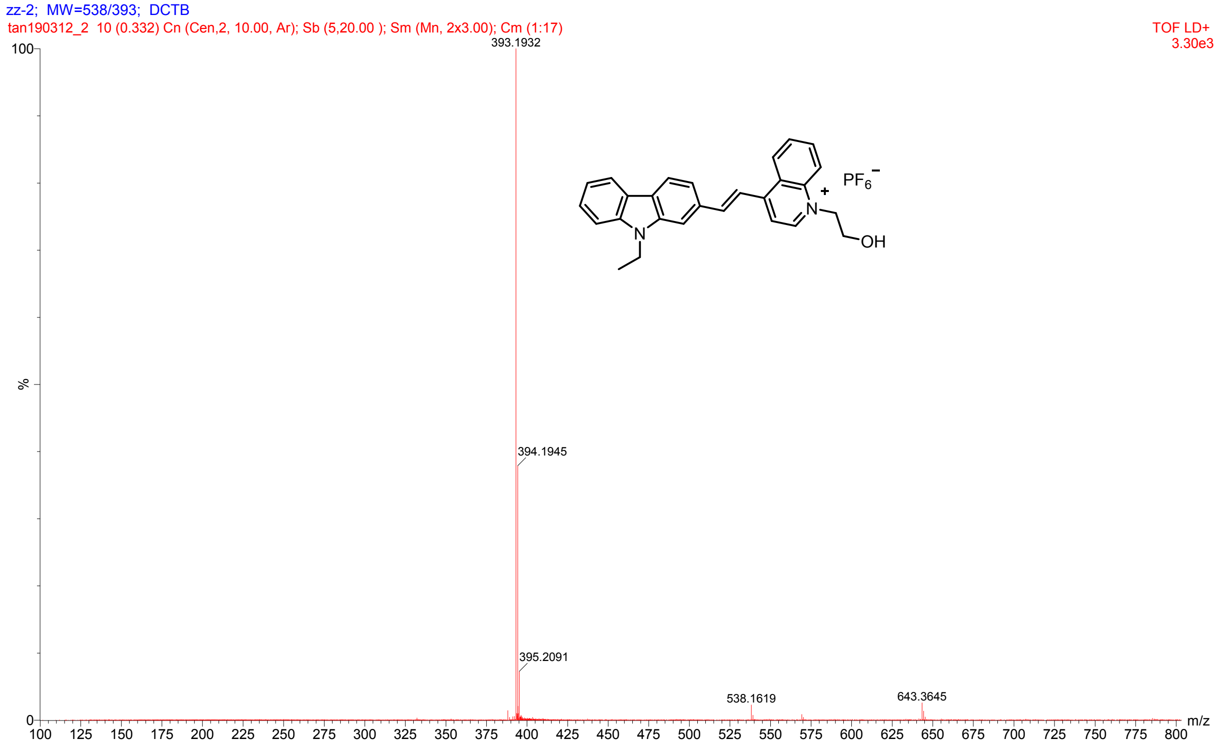

Figure S17. HRMS spectrum of CQu.

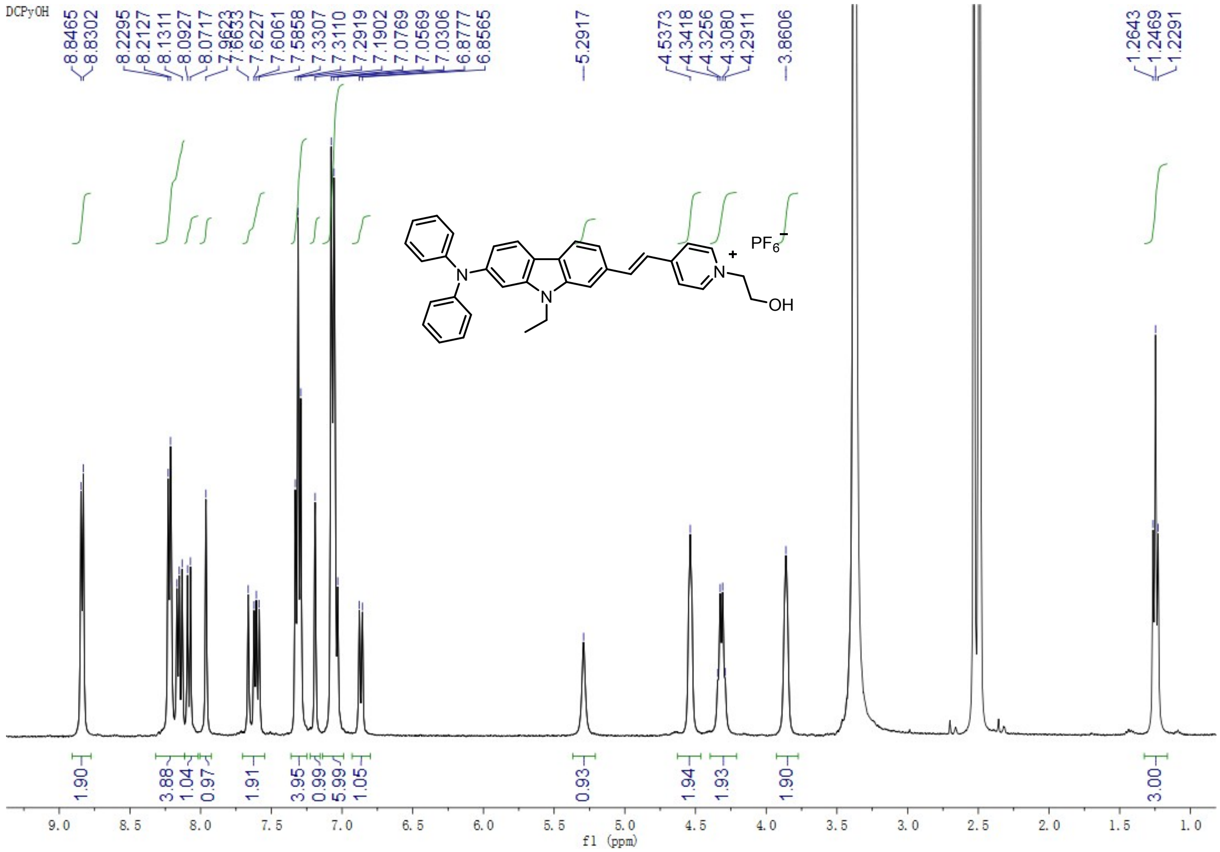

Figure S18. ^{1}H NMR spectrum of DCPy in DMSO-d_6.

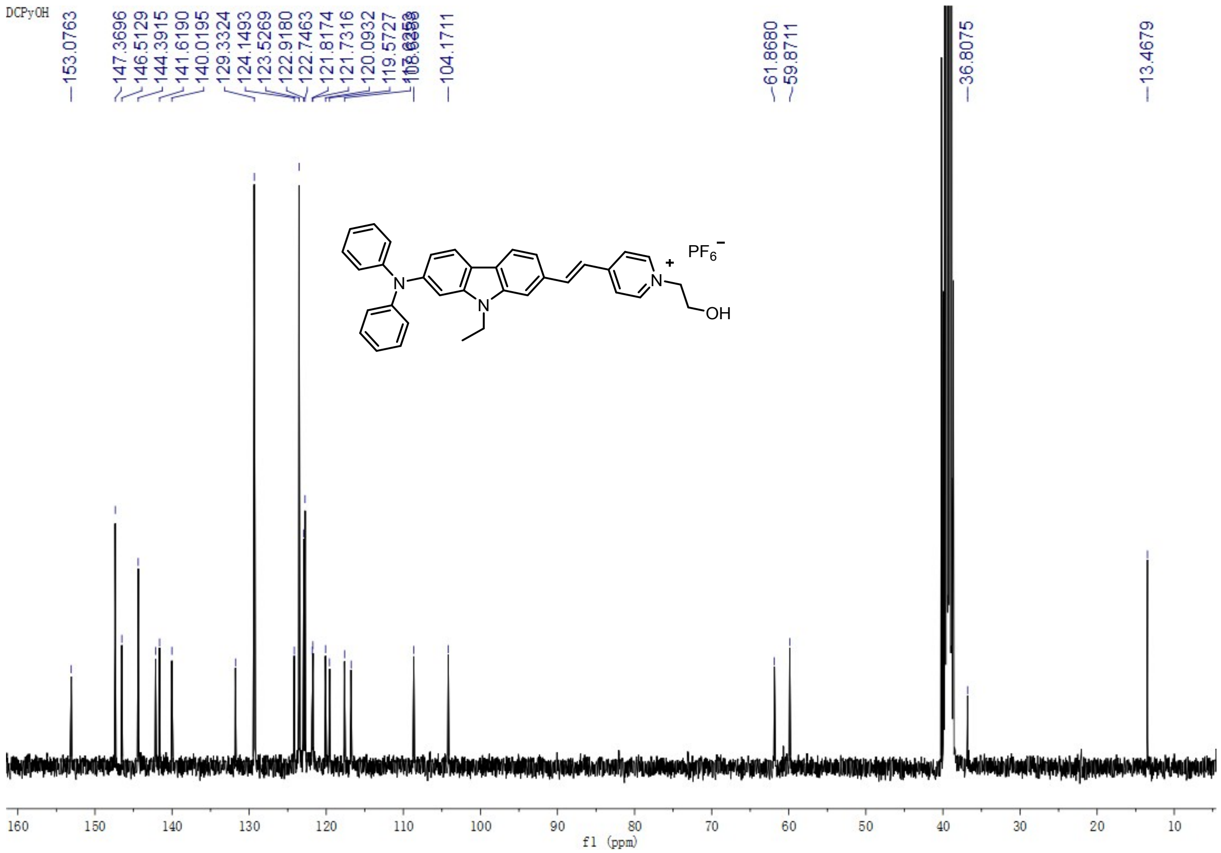

Figure S19. ^{13}C NMR spectrum of DCPy in DMSO-d_6.

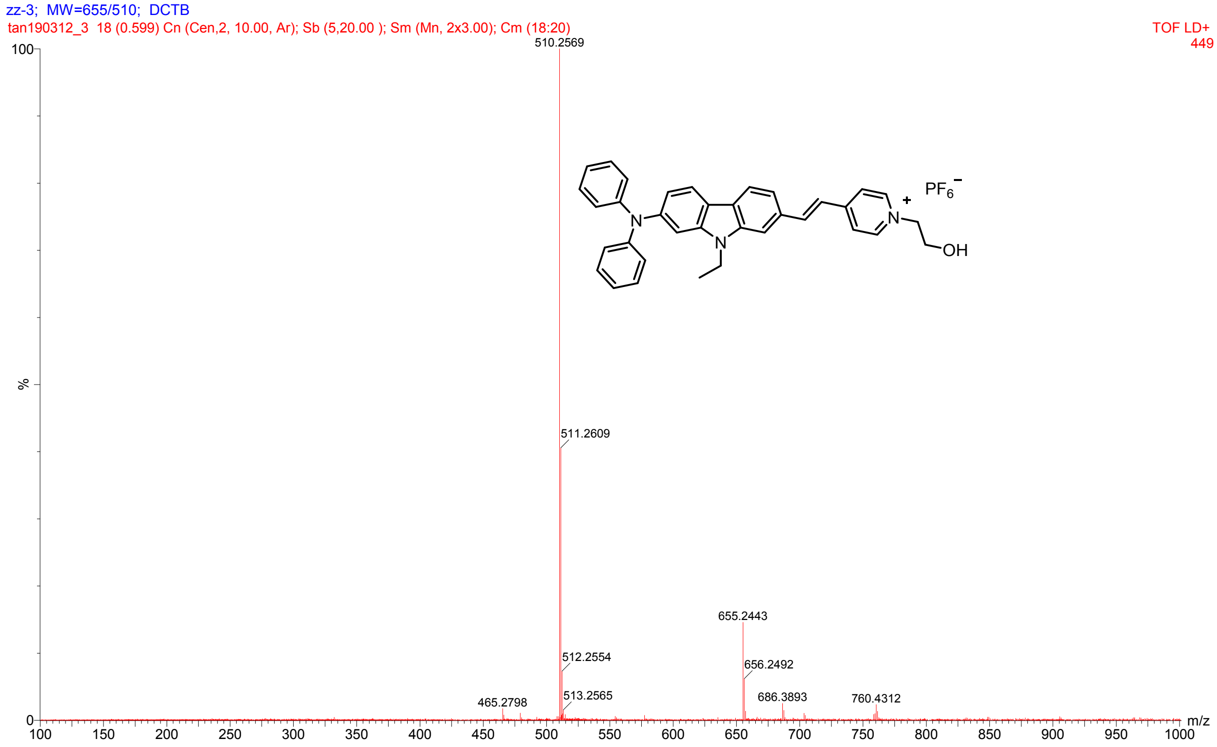

Figure S20. HRMS spectrum of DCPy.

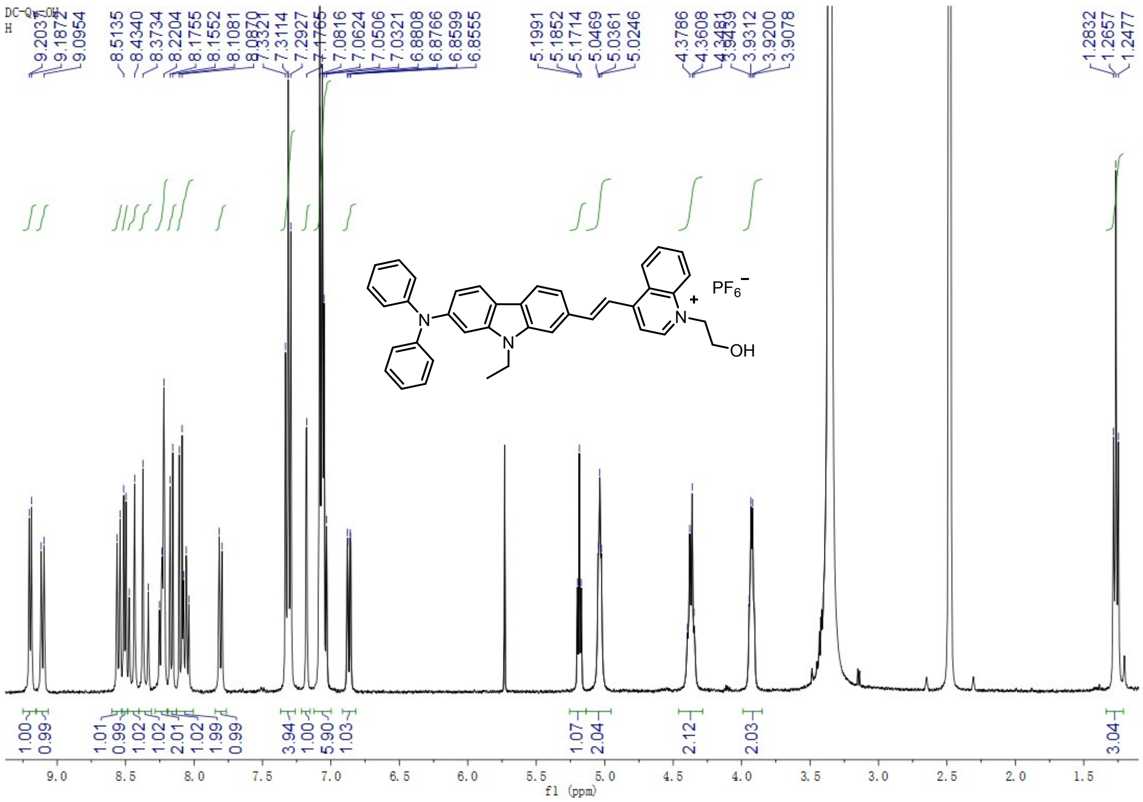

Figure S21. ^{1}H NMR spectrum of DCQu in DMSO-d_6.

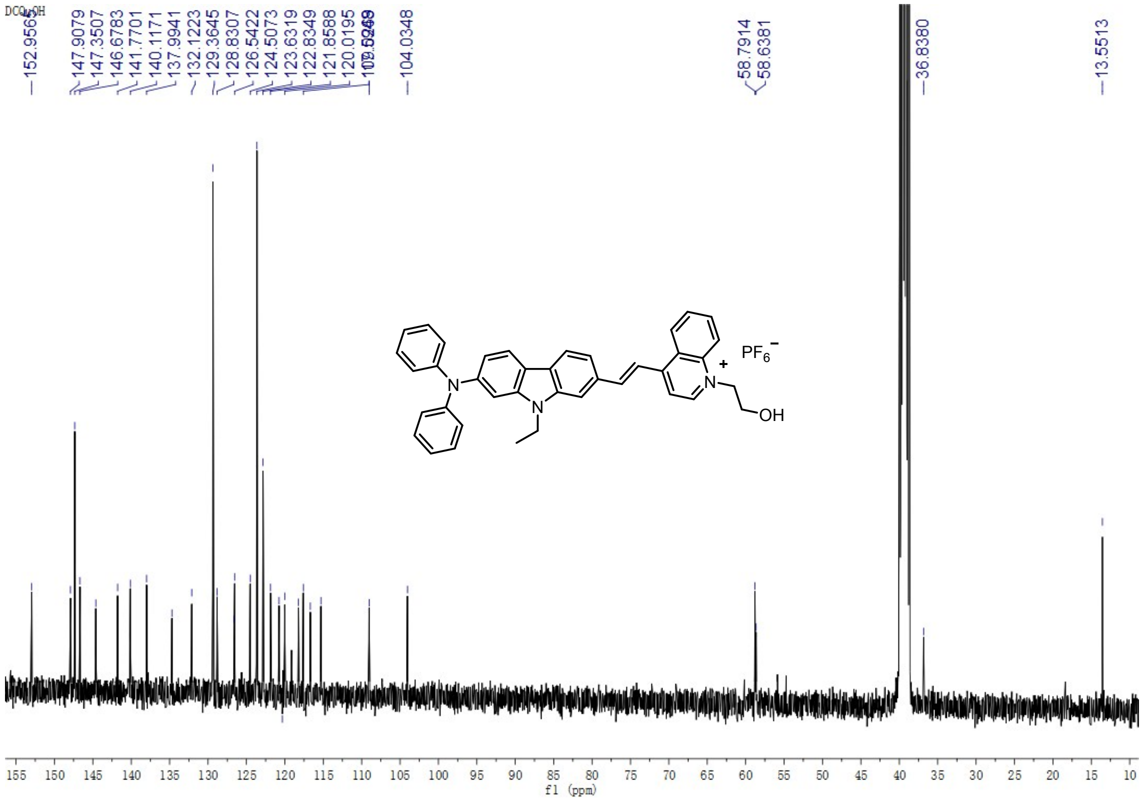

Figure S22. ^{13}C NMR spectrum of DCQu in DMSO-d_6.

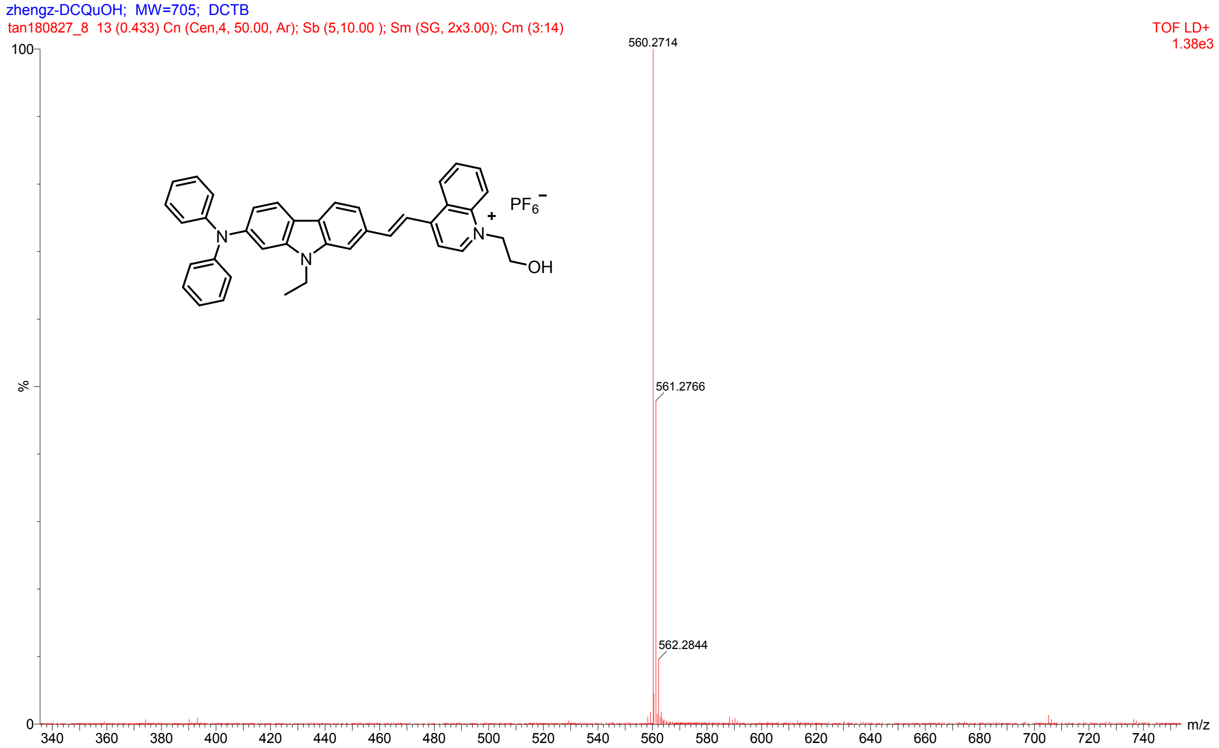

Figure S23. HRMS spectrum of DCQu.

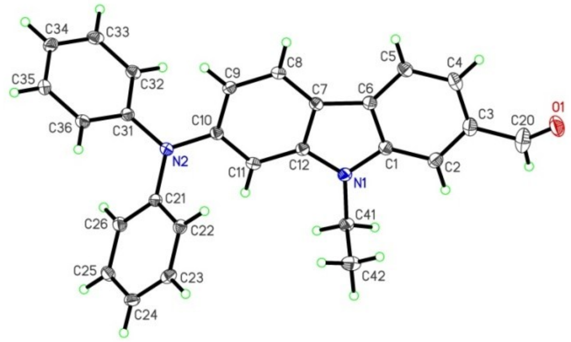

Table S1 Crystal data and structure refinement for intermediate aldehyde (CCDC: 1921647).

Identification code	zhengz1a
Empirical formula	$C_{27}H_{22}N_2O$
Formula weight	390.46
Temperature/K	100.01(10)
Crystal system	monoclinic
Space group	$P2_1/n$
a/Å	9.53897(10)
b/Å	23.1410(2)
c/Å	9.57210(10)
α/°	90
β/°	107.3297(12)
γ/°	90
Volume/Å^3	2017.04(4)
Z	4
ρ_{calc}g/cm^3	1.286
μ/mm^{-1}	0.613
F(000)	824.0
Crystal size/mm^3	0.3 × 0.3 × 0.25
Radiation	CuKα (λ = 1.54184)
2Θ range for data collection/°	7.64 to 134.97
Index ranges	$-11 \le h \le 11$, $-27 \le k \le 27$, $-11 \le l \le 7$
Reflections collected	10527
Independent reflections	3583 [R_{int} = 0.0108, R_{sigma} = 0.0110]
Data/restraints/parameters	3583/0/281
Completeness to theta = 66.5°	98.4%
Goodness-of-fit on F^2	1.001
Final R indexes [I>=2σ (I)]	R_1 = 0.0349, wR_2 = 0.0958
Final R indexes [all data]	R_1 = 0.0360, wR_2 = 0.0967
Largest diff. peak/hole / e Å^{-3}	0.20/-0.17

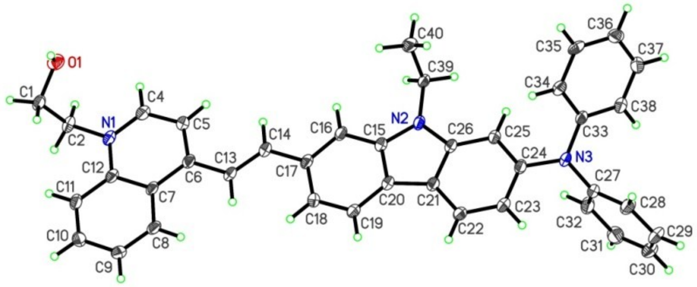

Table S2 Crystal data and structure refinement for DCQu (CCDC: 1921645).

Identification code	DCQu
Empirical formula	$C_{39}H_{34}F_6N_3OP$
Formula weight	705.66
Temperature/K	100.01(10)
Crystal system	monoclinic
Space group	$P2_1/c$
a/Å	9.3923(3)
b/Å	29.4208(8)
c/Å	11.9664(3)
α/°	90
β/°	102.511(3)
γ/°	90
Volume/Å^3	3228.14(16)
Z	4
ρ_{calc}g/cm^3	1.452
μ/mm^{-1}	1.390
F(000)	1464.0
Crystal size/mm^3	0.25 × 0.2 × 0.05
Radiation	CuKα (λ = 1.54184)
2Θ range for data collection/°	8.144 to 152.372
Index ranges	$-11 \leq h \leq 11$, $-36 \leq k \leq 23$, $-14 \leq l \leq 9$
Reflections collected	10260
Independent reflections	6341 [R_{int} = 0.0209, R_{sigma} = 0.0334]
Data/restraints/parameters	6341/0/453
Completeness to theta = 66.5°	98.1%
Goodness-of-fit on F^2	1.026
Final R indexes [I>=2σ (I)]	R_1 = 0.0650, wR_2 = 0.1706
Final R indexes [all data]	R_1 = 0.0750, wR_2 = 0.1803
Largest diff. peak/hole / e Å^{-3}	0.98/-0.52

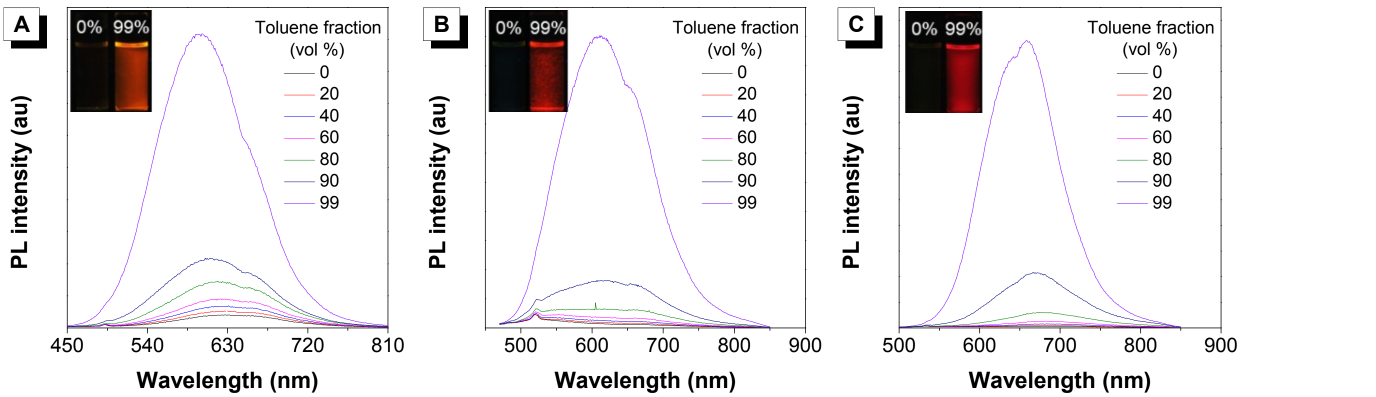

Figure S24. PL spectra of (A) CPy, (B) CQu, (C) DCPy in DMSO/toluene mixtures with different toluene fractions. [AIEgen] = 15 μM. Inset: fluorescent images of AIEgen (15 μM) in DMSO solution and in DMSO/toluene mixtures with 99% toluene fraction taken under 365 nm UV irradiation.

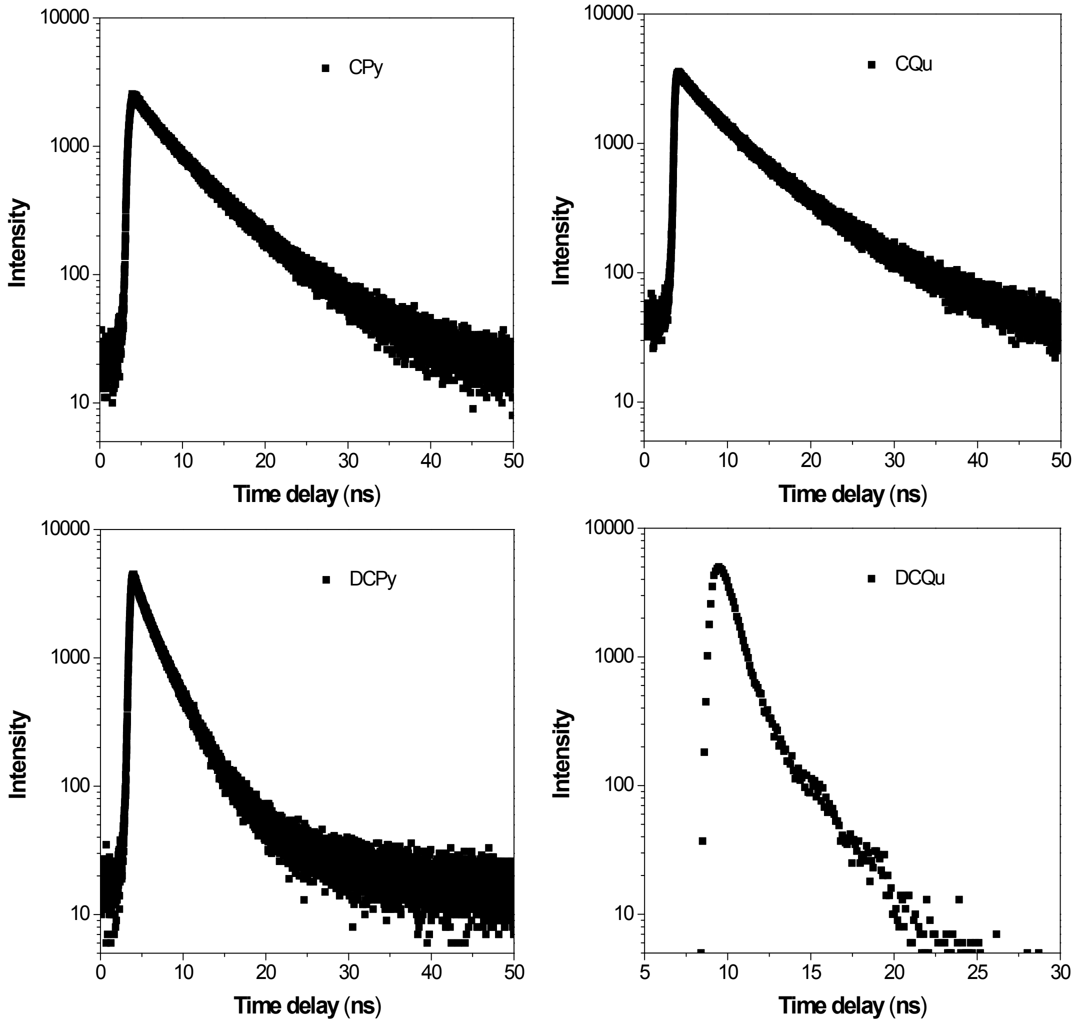

Figure S25. Fluorescence decay curves of CPy, CQu, DCPy and DCQu in solid state.

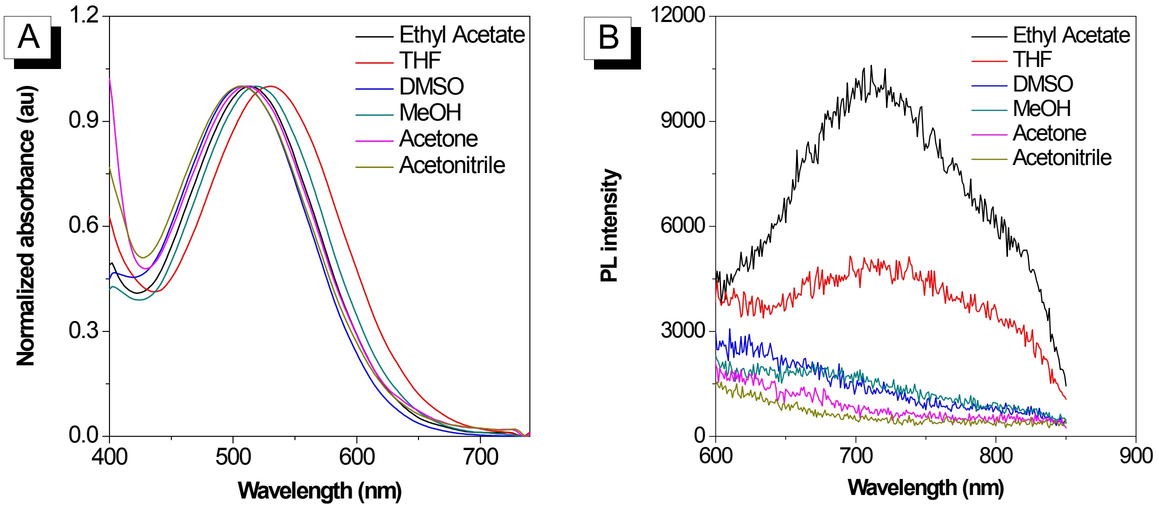

Figure S26. (A) Absorption and (B) PL spectra of DCQu in solvents with different polarities. Concentration: 10 µM; excitation wavelength: 500 nm.

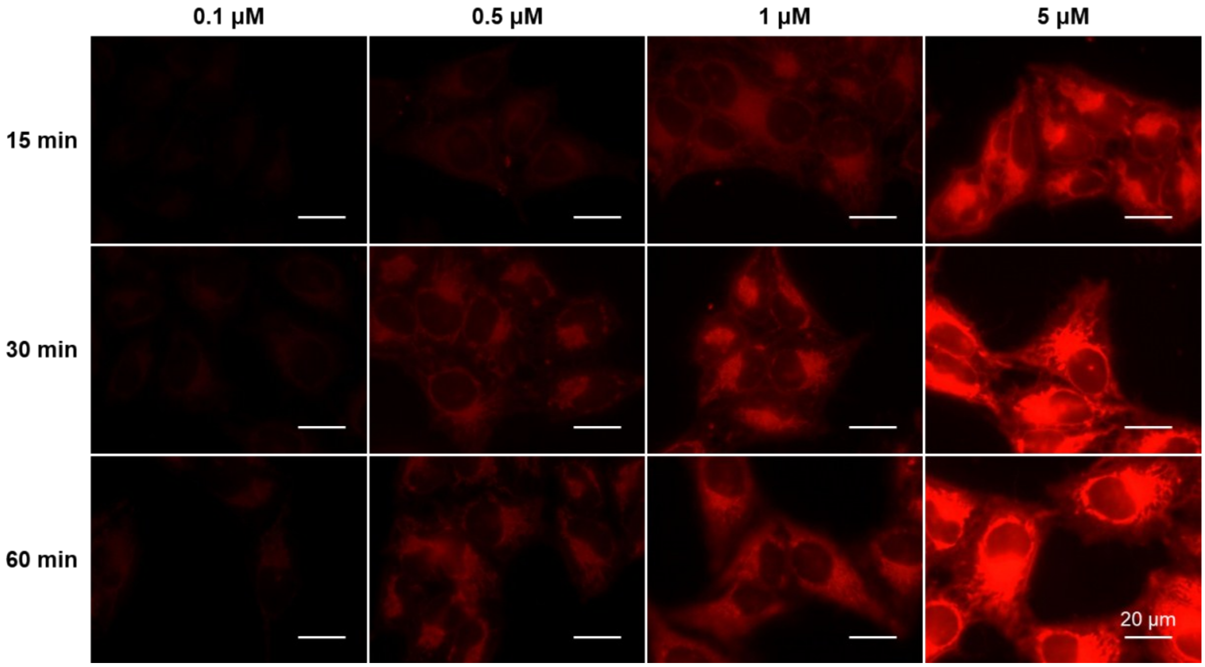

Figure S27. Fluorescent images of HeLa cells stained with different concentration of DCQu (0.1, 0.5, 1, and 5 µM) and with different incubation time (15, 30, and 60 min). Exposure time: 500 ms. Scale bar: 20 µm.

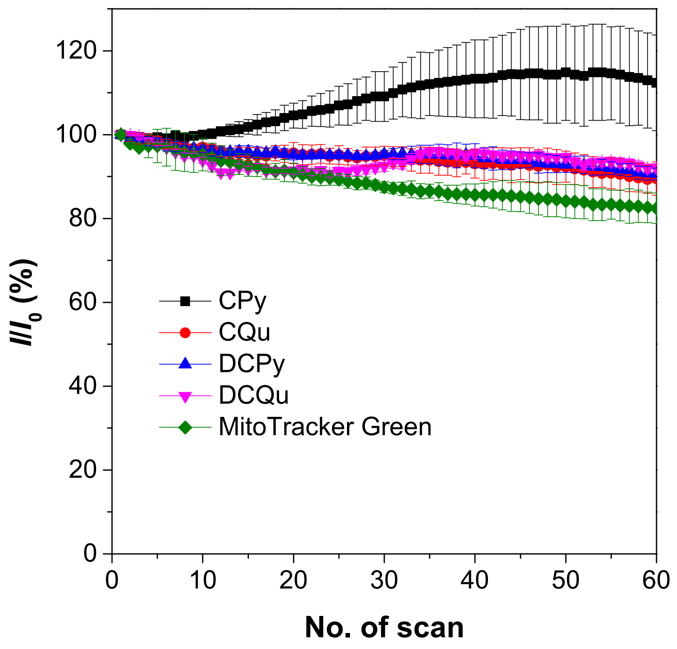

Figure S28. Loss in fluorescence of HeLa cells stained with CPy, CQu, DCPy, DCQu and MitoTracker Green with increasing number of sequential scans of laser irradiation. Emission signal was normalized to the maximum intensity at the beginning of irradiation.

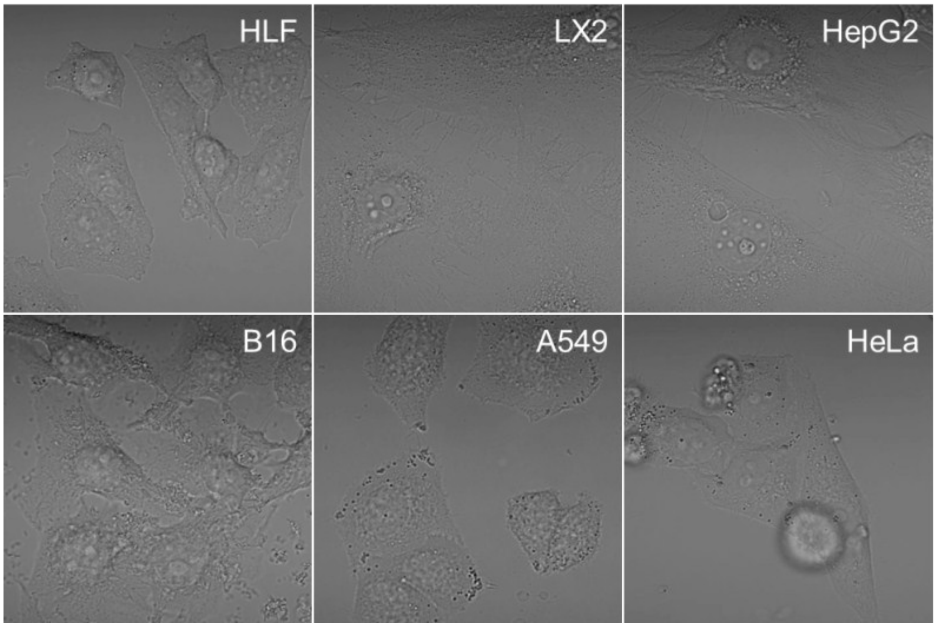

Figure S29. Bright-field image of different normal cells (HLF and LX2) and cancer cells (HepG2, B16, A549 and HeLa) stained with DCQu for 30 min. [DCQu] = 1 μM.

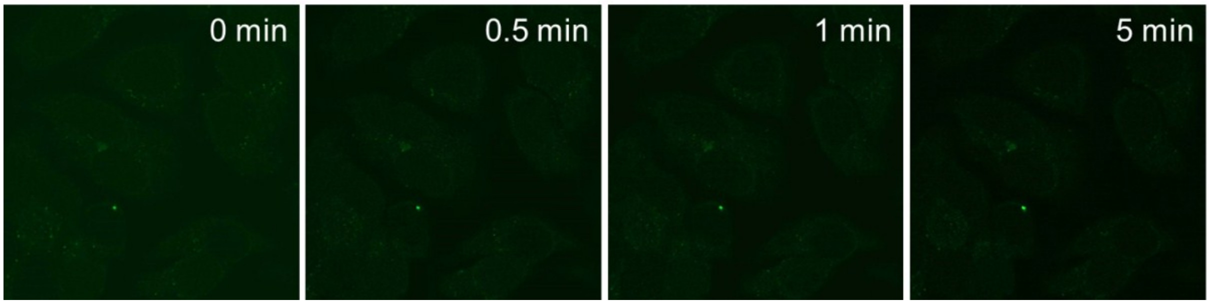

Figure S30. Detection of intracellular ROS generation using H2DCF-DA in HeLa cells incubated without DCQu followed by irradiation with white light irradiation for different time. Ex: 488 nm, Em: 580–740 nm.

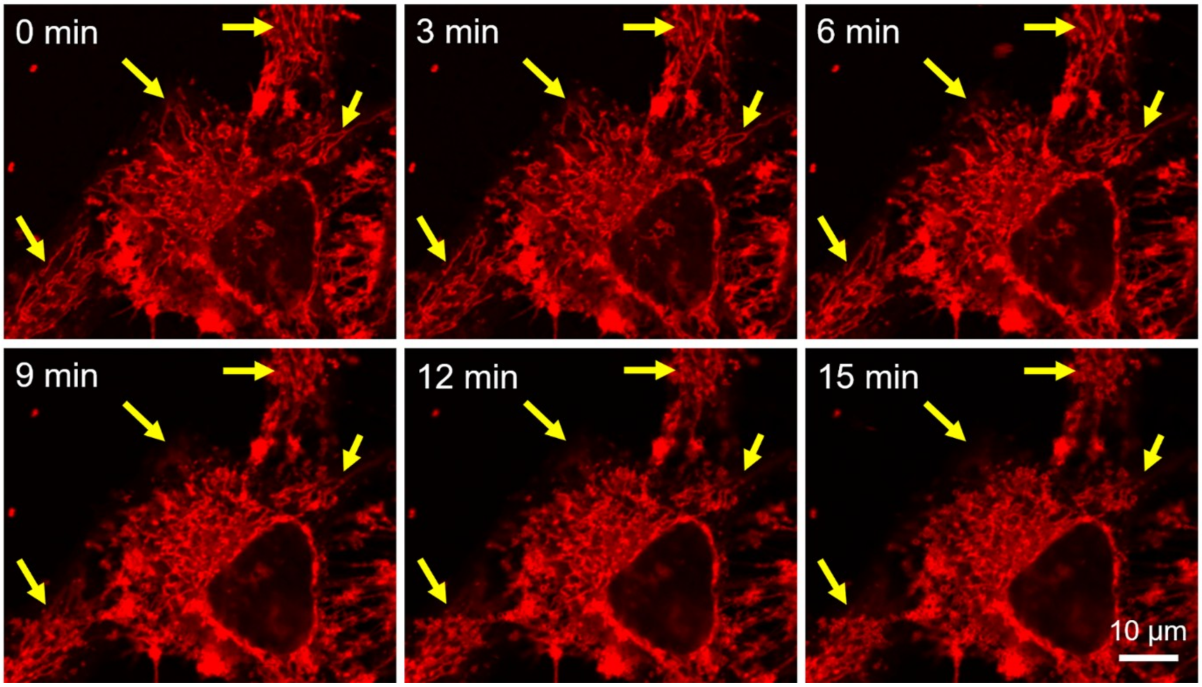

Figure S31. Monitoring the mitochondrial change during PDT by the fluorescence of DCQu. Fluorescence images of HeLa cells were incubated with 5 μM of DCQu for 30 min and then followed by continuous scanning for 15 min.

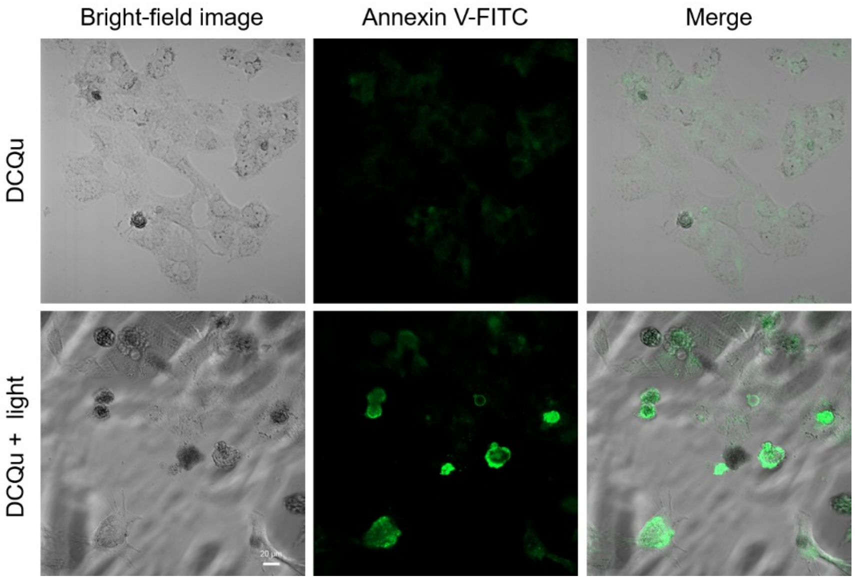

Figure S32. Annexin V-FITC target apoptotic cells after the PDT treatment.

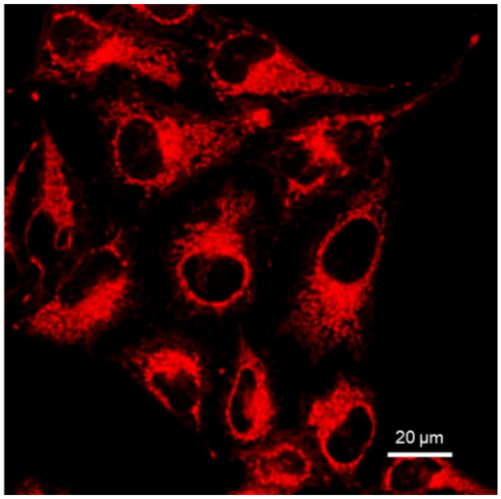

Figure S33. Two-photon excited fluorescence image of HeLa cells stained with DCQu for 30 min. [DCQu] = 5 μM. Two-photon excitation wavelength: 900 nm. Scale bar: 20 μm.

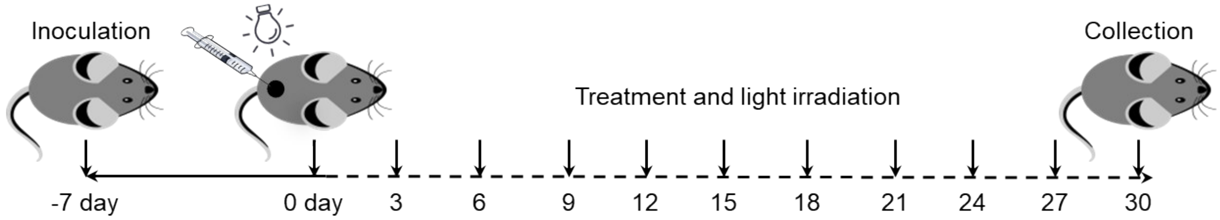

Figure S34. Diagram of *in vivo* PDT treatment of B16 melanoma-bearing mice with white light irradiation (4.2 mW cm^{-2}).

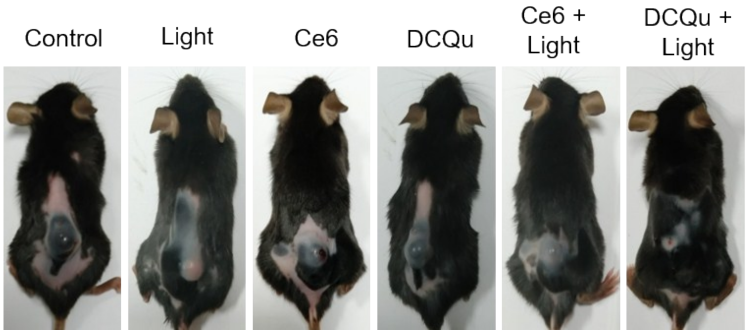

Figure S35. Representative images of B16 melanoma-bearing mice in different groups on 21 days during the treatment process.

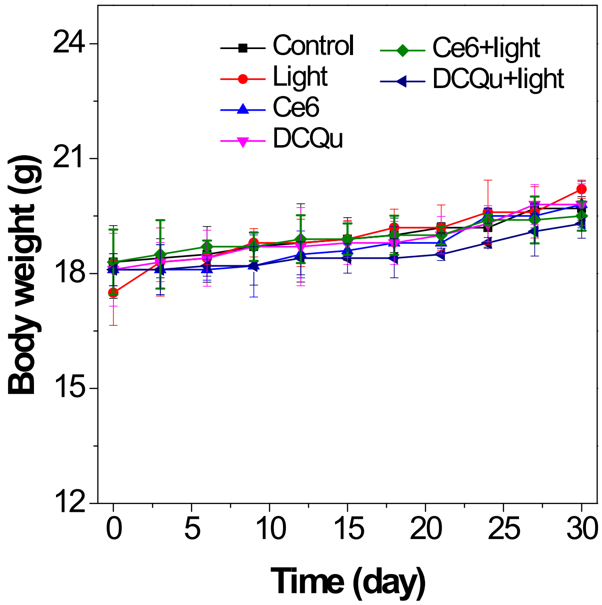

Figure S36. Body weights of B16 melanoma-bearing mice in different groups during the whole treatment period.

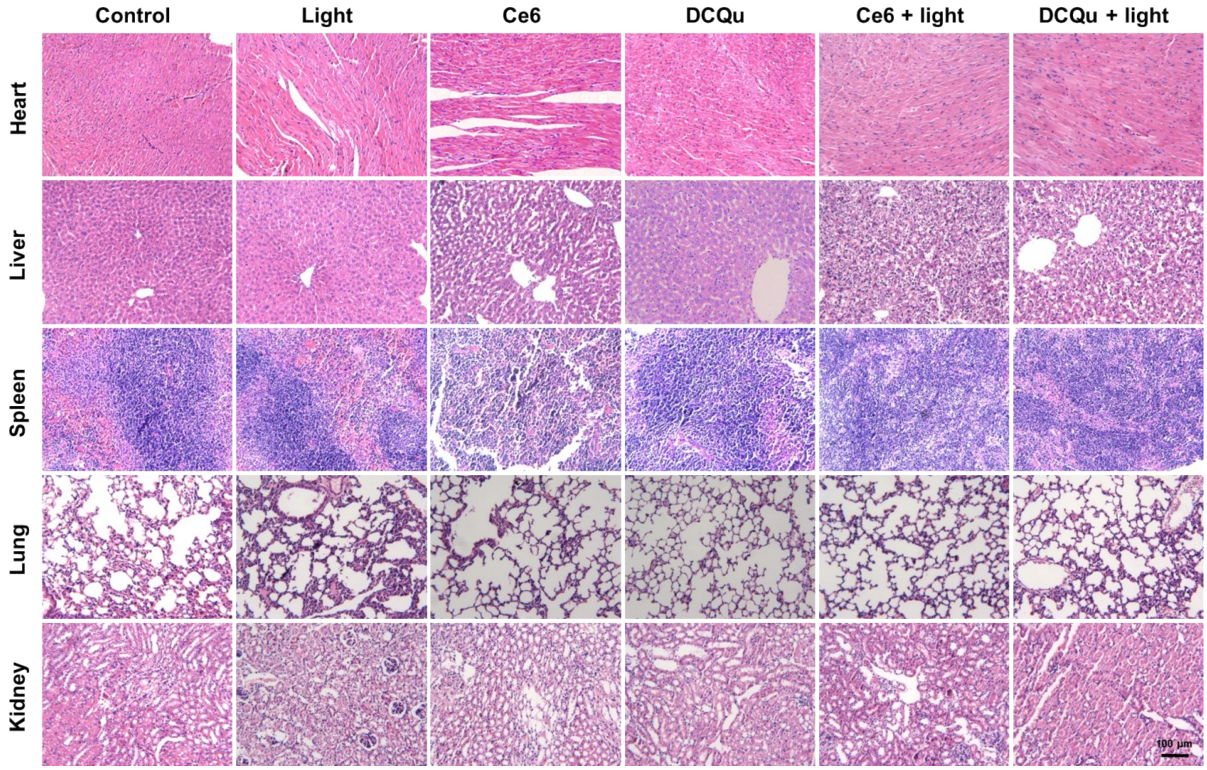

Figure S37. H&E-stained images of major organs of the mice after treating with light, free Ce6, free DCQu, Ce6 + light and DCQu + light. No noticeable abnormality was observed in major organs including heart, liver, spleen, lung, and kidney. Scale bar = 100 μm.
